# Infrared Spectra and Pressure-Dependent Yields of the Criegee Intermediate Methyl Vinyl Ketone Oxide and Its Iodoperoxy Adduct

**DOI:** 10.1021/acs.jpca.6c02328

**Published:** 2026-06-01

**Authors:** Ju-Yin Hsu, Yuan-Pern Lee

**Affiliations:** † Department of Applied Chemistry and Institute of Molecular Science, 34914 National Yang Ming Chiao Tung University, 1001, Ta-Hsueh Road, Hsinchu 300093, Taiwan; ‡ Center for Emergent Functional Matter Science, National Yang Ming Chiao Tung University, Hsinchu 300093, Taiwan

## Abstract

Methyl vinyl ketone oxide (MVKO), a key Criegee intermediate from isoprene ozonolysis, is produced in laboratories via ultraviolet photolysis of 1,3-diiodo-but-2-ene [(CH_2_I)­HC=C­(CH_3_)­I] in O_2_. While the 3-iodo-3-peroxylbut-1-ene [C_2_H_3_C­(CH_3_)­IOO, denoted IMVKO] adduct becomes stabilized at high pressures, the yield of MVKO decreases. We report the definitive infrared spectral identification of IMVKO, with seven observed bands showing excellent agreement with theoretical predictions. This observation also enables a more accurate MVKO spectrum, correcting prior reports that contained significant IMVKO contributions. By simultaneously monitoring IR bands of syn-MVKO and IMVKO, we quantified the pressure dependence of the syn-MVKO yield relative to the total of syn-MVKO and IMVKO, *y*_α_^rel^, to follow 1/*y*_α_^rel^ = (1.08 ± 0.11) + (1.08 ± 0.04)×10^–18^ [M]. Furthermore, incorporating quantum-chemically predicted IR intensities of MVKO and the loss of the precursor upon photolysis, the absolute yield (*y*_α_) of MVKO was estimated as 1/*y*_α_ = (2.74 ± 0.40) + (3.00 ± 0.13) × 10^–18^ [M]. Possible reasons for MVKO’s smaller yield than CH_2_OO are discussed.

## Graphical abstract

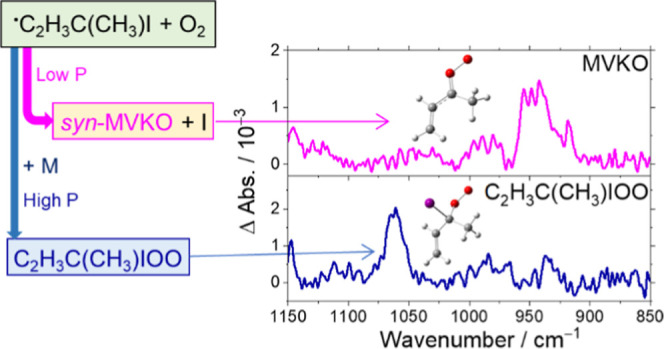

## 1 Introduction

The study of Criegee intermediates (CI) has expanded rapidly over the past decade, as documented in numerous review articles.^1–20^ Among these species, methyl vinyl ketone oxide [MVKO, CH_2_=CHC­(CH_3_)­OO] is of particular importance in the atmosphere because it is a primary product of isoprene ozonolysis.^21,22^ Isoprene is the most abundant nonmethane volatile organic compound (VOC) emitted into the atmosphere.^23,24^ The unique structure of MVKO, resonance-stabilized by the vinyl moiety, significantly influences its chemical reactivity.

Quantum-chemical calculations identified four stable conformers of MVKO: syn-*trans*, syn-*cis*, anti-*trans*, and anti-cis.^25,26^ Here, syn and anti denote the orientation of the terminal O atom relative to the methyl moiety, while *cis* and *trans* describe the relative orientation of the C=C and C=O bonds. The syn-*trans*-MVKO conformer has the lowest energy, with the syn-*cis*-, anti-*trans*-, and anti-*cis*-MVKO lying ∼7, 11, and 12 kJ mol^–1^ higher in energy, respectively. While the *cis*- and *trans*-conformers equilibrate at ambient temperature due to a relatively low interconversion barrier (∼40 kJ mol^–1^),^27,28^ the anti- and syn-MVKO are considered distinct chemical entities owing to a much higher interconversion barrier of ∼120 kJ mol^–1^.

A novel method for laboratory synthesis of MVKO was reported by Barber et al., utilizing photolysis of a gaseous mixture of 1,3-diiodobut-2-ene [(CH_2_I)­HC=C­(CH_3_)­I] and O_2_ at 248 nm.^25^ In this process, UV irradiation preferentially cleaves the terminal allylic C–I bond over the vinylic C–I bond to produce the 3-iodo-but-2-en-1-yl radical [^•^C_2_H_3_C­(CH_3_)­I].^29,30^ Subsequent reaction with O_2_, followed by the elimination of the remaining iodine atom, yields MVKO.

The spectral characterization of MVKO has been pursued through several experimental techniques. Endo et al. recorded the rotational spectrum of MVKO, though only the syn-*trans*-MVKO conformer was characterized.^31^ Chung and Lee reported the transient infrared (IR) absorption spectrum of MVKO at 35 Torr, assigning the primary features to syn-*trans*-MVKO and the weaker bands to, tentatively, syn-*cis*-MVKO.^29^ Barber et al. reported IR action spectra (5750–6300 cm^–1^) via laser-induced fluorescence detection of the OH product; while these features were attributed to both syn- and anti-MVKO, specific conformer assignments remained elusive.^25^ Electronic spectroscopy provides further information. Vansco et al. recorded the UV spectrum of jet-cooled MVKO by monitoring the decrease in MVKO ions (*m*/*z* = 86), produced upon ionization at 10.5 eV, during UV irradiation.^32^ The spectrum of MVKO shows an asymmetric feature spanning 300–450 nm with a maximum near 388 nm. In contrast, measurements at ambient temperature by Caravan et al.^27^ and Lin et al.^33^ revealed a more symmetric band centered near 370.6 nm. The discrepancy between jet-cooled and ambient spectra is thought to arise from the rapid unimolecular decay of anti-MVKO at room temperature. Although the UV spectrum of anti-MVKO is predicted to be red-shifted relative to syn-MVKO,^32,34–36^ it has yet to be experimentally characterized. Furthermore, the absolute absorption cross-section of syn-MVKO (299 K) was determined by Lin et al. to be (3.02 ± 0.60) × 10^–17^ cm^2^ molecule^–1^ at 352 nm, assuming thermal equilibrium between the syn-trans- and syn-cis-species; a maximal cross-section of (3.70 ± 0.74) × 10^–17^ cm^2^ molecule^–1^ at 371 nm was deduced from the reported UV spectrum and the cross-section at 352 nm.^37^

The reaction between the^•^C_2_H_3_C­(CH_3_)I and O_2_ is expected to proceed via four possible channels

1a C2•H3C(CH3)I+O2→syn‐MVKO+I

1b C2•H3C(CH3)I+O2→anti‐MVKO+I

1c C2•H3C(CH3)I+O2+M→C2H3C(CH3)IOO+M

1d C2•H3C(CH3)I+O2→others

in which *y*_α_, *y*_β_, *y*_γ_, and *y*_δ_ represent the respective yields, and C_2_H_3_C­(CH_3_)­IOO (hereafter denoted IMVKO) represents the stabilized iodoperoxy adduct, 3-iodo-3-peroxylbut-1-ene. Lin et al. observed a gradual increase in MVKO concentration over several ms at high pressures, which they attributed to the dissociation of IMVKO into syn-MVKO + I^33^

2 $$C_{2}H_{3}C\left({CH}_{3}\right)IOO+M\to sy\mathrm{n‐}MVKO+I+M$$

Lin et al. reported a rate coefficient of *k*_2_ ∼ 1100 s^–1^ for reaction (2) at 298 K; the temperature dependence of *k*_2_ over the range 278–319 K yielded an activation energy of 53 ± 1 kJ mol^–1^, consistent with the calculated dissociation energy of 59 kJ mol^–1^ for reaction (2). These authors further reported that the relative yield of IMVKO, *y*_γ_/(*y*_α_ + *y*_γ_), is strongly pressure-dependent, increasing from ∼0.25 below 10 Torr to ∼0.85 above 300 Torr.^33^ However, the combined yield of syn-MVKO and IMVKO, *y*_α_ + *y*_γ_, was reported to be 0.22 ± 0.10 at 299 K over the pressure range 30–700 Torr;^37^ this combined yield is notably small and poorly constrained due to the large error bars.

The UV spectrum of anti-MVKO has not been reported experimentally, likely due to its rapid unimolecular isomerization, with a predicted rate coefficient of 2100–64000 s^–1^ at 1 atm and 298 K.^18,25,27,28^ The unimolecular decay pathway proceeds via a dioxole intermediate, *cyc*-(CH_3_)­[C=CHCH_2_OO], to produce acetyl (CH_3_^•^CO) and vinoxy (CH_2_CH^•^O) radicals.^18,25,26^ Conversely, the unimolecular decomposition of syn-MVKO is much slower, with predicted rate coefficients of 24–102 s^–1^ (1 atm, 298 K)^18,25,27,37^ and an experimental value of 70 s^–1^.^38^ Evidence for the rapid decay of anti-MVKO was provided by Vansco et al., who detected secondary reaction products, such as formaldehyde (88 ± 5%), ketene, and glyoxal, at a total pressure of 10 Torr and ambient temperature, using multiplexed photoionization mass spectrometry (MPIMS).^39^ These stable products were predicted to originate from the reaction of acetyl and vinoxy radicals with O_2_.^40,41^

In this work, we present the definitive IR characterization of IMVKO. The pressure-dependent yield of syn-MVKO relative to IMVKO was determined by simultaneously monitoring IR bands of syn-MVKO and IMVKO. We further estimated the absolute yield of syn-MVKO to be higher than previously reported. Through these analyses, we also found that prior IR spectra of MVKO at 35 Torr^29^ were contaminated by IMVKO.

## 2 Methods

Transient infrared absorption spectra were recorded using a step-scan Fourier-transform infrared (FTIR) spectrometer, as described previously.^42,43^ Photolysis of the (*Z*)-(CH_2_I)­HC=C­(CH_3_)I precursor within a White cell reactor (path length 3.6 m, volume 1370 cm^3^) was initiated by a KrF excimer laser (248 nm, 4–8 Hz, 185 mJ pulse^–1^, beam size 10.2 cm^2^). The IR probe beam was detected by an HgCdTe detector (77 K) using ac- and dc-coupled outputs, digitized with a 14 bit ADC at 4 ns resolution. A total of 9500 data points were acquired over a 38 μs reaction period, including ∼2 μs before photolysis. To improve signal-to-noise ratios (SNR), the signals were averaged over 15 laser shots at each step-scan position; 4–7 spectra recorded under similar experimental conditions were further averaged. Under-sampling, coupled with optical filters, was employed to minimize data-acquisition time. For the spectral range 850–1450 cm^–1^ at an instrumental resolution of 2 cm^–1^, a total of 846 step-scan positions were required.

Gaseous (*Z*)-(CH_2_I)­HC=C­(CH_3_)­I was carried into the reactor via N_2_ or O_2_ streams. The flow rates of O_2_ and N_2_ were 2.3–23.3 and 2.0–26.7 STP cm^3^ s^–1^, respectively. Experiments were conducted at 298 K across a pressure range of 15–194 Torr. The photolysis efficiency was estimated at ∼10% based on a laser fluence of ∼7.7 × 10^16^ photons cm^–2^. Precursor partial pressures were determined via Beer’s law using measured IR cross sections in the 1120–1210 and 1265–1320 cm^–1^ regions. (*Z*)-(CH_2_I)­HC=C­(CH_3_)I (>95%, Accela), N_2_ (99.9995%, Chiah-Lung), and O_2_ (99.99%, Chiah-Lung) were used as received.

Quantum-chemical calculations were performed using Gaussian 16.^44^ Geometries, harmonic vibrational wavenumbers, and IR intensities were calculated at the B3LYP/aug-cc-pVTZ level of theory. The B3LYP density-functional theory (DFT) uses Becke’s three-parameter hybrid exchange functional with a correlation functional of Lee et al.^45–47^ and Dunning′s correlation-consistent polarized-valence triple-ζ basis set, augmented with s, p, d functions (aug-cc-pVTZ).^48,49^ For the iodine atom, the aug-cc-pVTZ-pp basis set was employed to account for the relativistic effects of the heavy element.^50^

## 3 Results and Discussion

### 3.1 Transient IR Spectra of MVKO and IMVKO

Transient IR spectra were recorded with a step-scan Fourier-transform infrared (FTIR) spectrometer at a resolution of 2 cm^–1^. The partial IR absorption spectrum, recorded 0–10 and 10–20 μs following photolysis at 248 nm of a flowing mixture of (*Z*)-(CH_2_I)­HC=C­(CH_3_)­I/O_2_ (0.02/14.9 Torr) at 298 K, is shown in Figure 1. New absorption features were observed near 1110, 986, and 945 cm^–1^, while downward features at 1434, 1294, 1152, and 1061 cm^–1^ corresponded to the depletion of the precursor (CH_2_I)­HC=C­(CH_3_)­I. The spectra were processed by adding back the precursor consumption and removing features associated with the radical precursor ^•^C_2_H_3_C­(CH_3_)I and methyl vinyl ketone (MVK); the results are shown in Figure 1d,e, respectively. The processed spectrum for the 10–20 μs intervals at 14.9 Torr is also presented in Figure 2a. Similar experiments were conducted at a higher total pressure of 194 Torr using a mixture of (*Z*)-(CH_2_I)­HC=C­(CH_3_)­I/O_2_/N_2_ (0.05/15/179 Torr) at 298 K. The raw and processed spectra are displayed in Figure S1; the processed spectrum (at 194 Torr) covering 10–20 μs is shown in Figure 2b. Notably, the feature near 1060 cm^–1^ is significantly enhanced at 194 Torr, suggesting the stabilization of a second species. To isolate the low-pressure component (predominantly MVKO), we performed a spectral subtraction (Figure 2a–0.25 × Figure 2b), yielding the spectrum in Figure 2c (labeled B_1_–B_7_). The 0.25 factor was chosen to achieve a near-zero baseline in the 1040–1080 cm^–1^ region. Conversely, to isolate the high-pressure component, we subtracted 0.53 × Figure 2a from Figure 2b, revealing new features labeled C_1_–C_7_ (Figure 2f). The 0.53 factor was determined by the disappearance of the broad band of MVKO near 945 cm^–1^.

**Figure 1:**
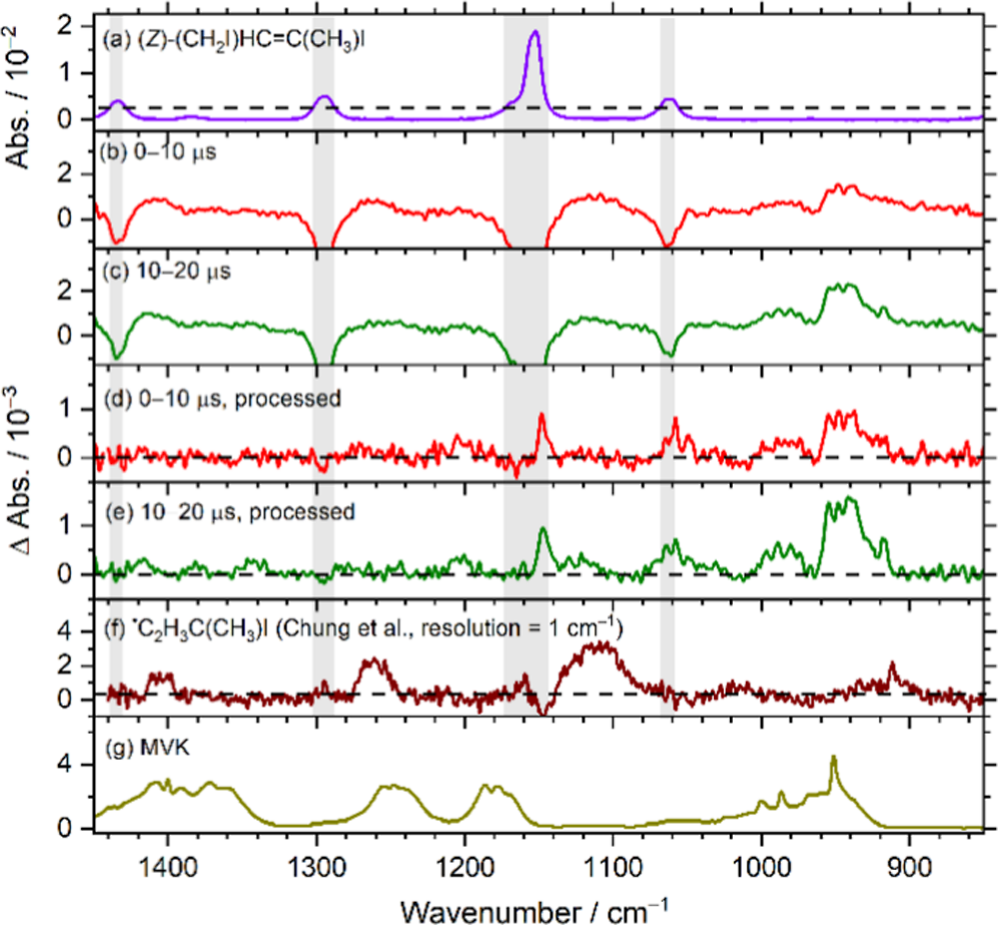
Observed and processed spectra in the region 1450–850 cm^–1^ upon photolysis at 248 nm of a flowing mixture of (*Z*)-(CH_2_I)­HC=C­(CH_3_)­I/O_2_ (0.02/14.9 Torr). (a) Absorption spectrum before photolysis. (b,c): Difference spectra recorded 0–10 μs (b) and 10–20 μs (c) after photolysis. (d,e): Processed spectra of (b,c) by removing bands of the radical precursor ^•^C_2_H_3_C­(CH_3_)I and methyl vinyl ketone (MVK), and adding back the decay of (*Z*)-(CH_2_I)­HC=C­(CH_3_)­I. Gray areas indicate severe interference from the absorption of (*Z*)-(CH_2_I)­HC=C­(CH_3_)­I. The instrumental resolution is 2 cm^–1^. An external ADC was employed. (f) Spectrum of radical precursor ^•^C_2_H_3_C­(CH_3_)I reported in Chung and Lee.^29^ (g) Reference spectrum of methyl vinyl ketone (MVK) at 15 Torr. The negative bands of (*Z*)-(CH_2_I)­HC=C­(CH_3_)I in (a,b) are truncated. Figure 1f was reproduced with permission from ref 29. Copyright 2021, http://creativecommons.org/licenses/by/4.0/, Springer Nature.

**Figure 2:**
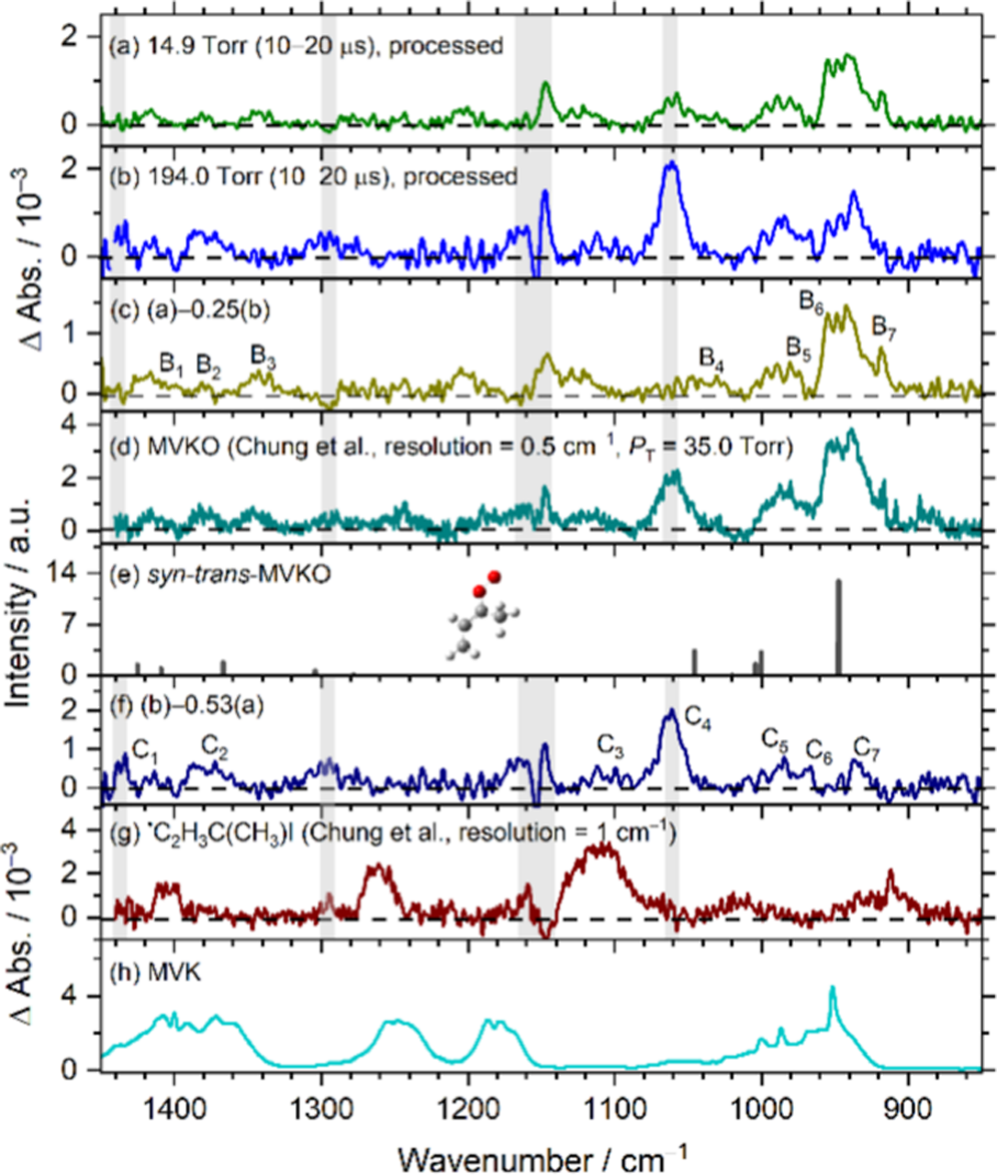
Processed spectra in the region 850–1450 cm^–1^ recorded 10–20 μs after photolysis at 248 nm of a flowing mixture of (*Z*)-(CH_2_I)­HC=C­(CH_3_)­I/O_2_/N_2_. The instrumental resolution is 2 cm^–1^. (a,b): Processed spectra recorded at *P*_T_ = 14.9 Torr (a) and *P*_T_ = 194.0 Torr (b), taken from Figures 1e and S1e, respectively. Spectra were processed by removing bands of radical ^•^C_2_H_3_C­(CH_3_)I and methyl vinyl ketone (MVK), and adding back the decay of (*Z*)-(CH_2_I)­HC=C­(CH_3_)­I. (c) Difference spectrum from spectrum in (a) minus 0.25 times that in (b). Bands in group B are labeled B_1_–B_7_. (d) Reference spectra of syn-MVKO.^29^ (e) IR stick spectrum of syn-*trans*-MVKO according to scaled harmonic vibrational wavenumbers and IR intensities calculated with the B3LYP/aug-cc-pVTZ method.^29^ (f) Difference spectrum from spectrum in (b) minus 0.53 times that in (a). Bands in group C are labeled C_1_–C_7_. (g) Reference spectrum of radical precursor ^•^C_2_H_3_C­(CH_3_)I reported in ref 29. (h) Reference spectrum of methyl vinyl ketone (MVK) at 15 Torr. Figures 2d,g were reproduced with permission from ref 29. Copyright 2021, http://creativecommons.org/licenses/by/4.0/, Springer Nature.

The low-pressure bands B_1_–B_7_ (Figure 2c) correspond to MVKO, which was predominantly produced from ^•^C_2_H_3_C­(CH_3_)I + O_2_ at low pressure. For comparison, Figure 2d shows the MVKO spectrum previously reported by Chung and Lee at 35 Torr (0.5 cm^–1^ resolution),^29^ alongside the scaled harmonic stick spectrum of syn-*trans*-MVKO calculated at the B3LYP/aug-cc-pVTZ level of theory (Figure 2e). While our MVKO spectrum generally agrees with the literature, the band previously assigned to MVKO near 1060 cm^–1^ is significantly weaker in our processed spectrum and is instead located at 1038 cm^–1^. This difference is critical; as discussed below, the feature at 1060 cm^–1^ observed at 35 Torr by Chung and Lee contains a non-negligible contribution from the stabilized IMVKO adduct. The weaker feature, newly identified at 1038 cm^–1^ in this work, aligns more accurately with the scaled harmonic vibrational wavenumber of 1045 cm^–1^ for *syn-trans*-MVKO.

The dominant features observed at high pressure (group C, Figure 2f, reproduced in Figure 3a) are attributed to the C_2_H_3_C­(CH_3_)­IOO (IMVKO) adduct. Quantum-chemical calculations indicate the existence of nine IMVKO conformers, with the three lowest-energy structures differing by less than 1 kJ mol^–1^; others are higher in energy by more than 3.1 kJ mol^–1^.^29^ The simulated IR spectra of these conformers, using scaled harmonic vibrational wavenumbers and IR intensities (Table S1), are depicted in Figure S2b–d. A Boltzmann-weighted convolution of these species (conformer 1:2:3 = 40:31:29) at 298 K with a full width at half-maximum (fwhm) of 4 cm^–1^, is presented in Figure S2e and reproduced in Figure 3b. Other conformers are not included because they have a Boltzmann population of <10% of all conformers at 298 K. The fwhm was chosen based on empirical similarity with experiments. Changing the fwhm to 3 or 6 cm^–1^ did not significantly affect the overall characteristic of the spectrum.

**Figure 3:**
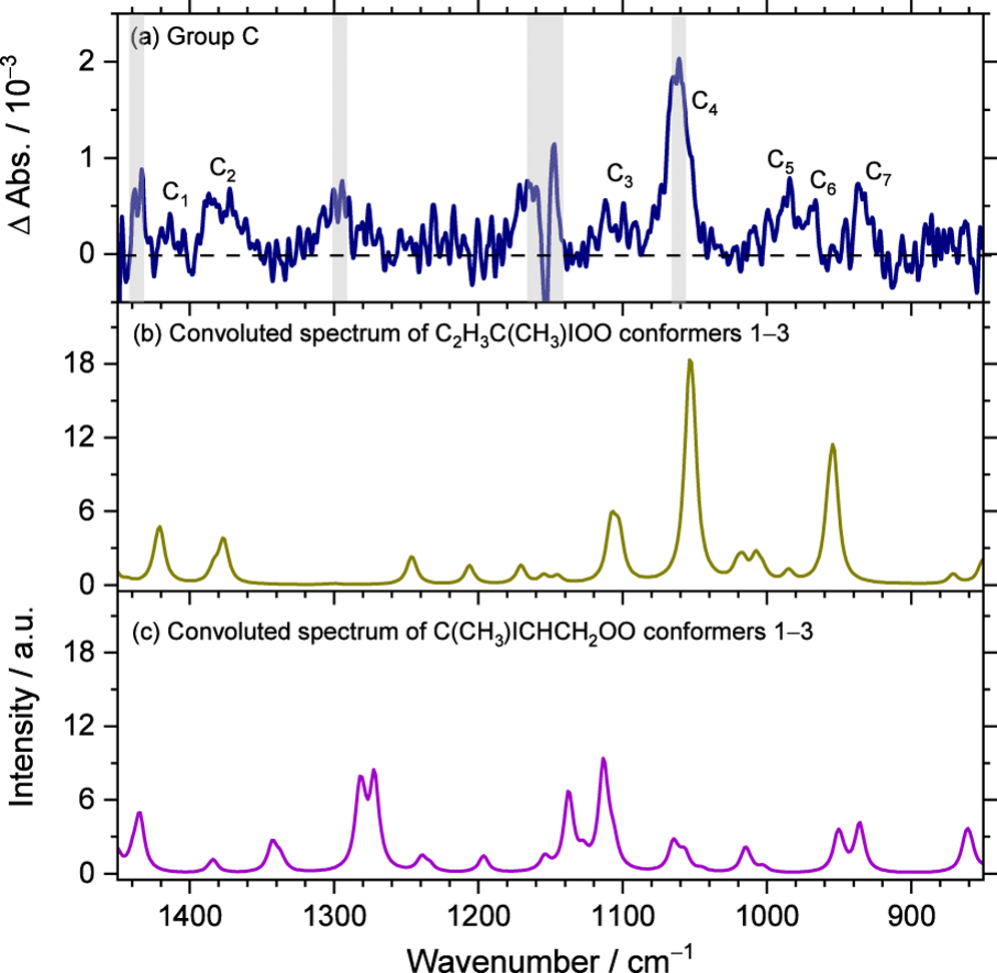
Comparison of bands in group C with the Boltzmann-weighted convoluted spectrum of two isomers of iodoperoxy adducts. (a) Processed spectrum of group C (from Figure 2f). Gray areas indicate spectral regions severely interfered with by precursor absorption. (b) Convoluted spectrum of three lowest-energy conformers of C_2_H_3_C­(CH_3_)­IOO with Boltzmann population distribution of 40:31:29; taken from Figure S2e. (c) Convoluted spectrum of three lowest-energy conformers of C­(CH_3_)­ICHCH_2_OO with Boltzmann population distribution of 48:36:16, taken from Figure S3e. For each isomer, scaled harmonic vibrational wavenumbers and IR intensities were predicted with the B3LYP/aug-cc-pVTZ-pp method.

The observed bands in group C exhibit satisfactory agreement with the predicted vibrational wavenumbers and IR intensities for IMVKO. A detailed comparison of values of these features with the scaled harmonic vibrational wavenumbers and relative IR intensities predicted for conformers 1–3 of IMVKO is summarized in Table S2. A comparison of observed values with the Boltzmann-weighted convoluted peak positions and average intensities is shown in Table 1. Specifically, the observed C_1_–C_7_ bands at 1435, 1371, 1105, 1060, 986, 964, and 931 cm^–1^ correspond well to the convoluted peaks of C_2_H_3_C­(CH_3_)­IOO at 1421, 1376, 1108, 1054, 1018, 985, and 954 cm^–1^, respectively. Notably, all bands in this region with IR intensities greater than 8 km mol^–1^ have been observed. The mean absolute deviation between experiments and convoluted scaled harmonic vibrational wavenumbers of IMVKO is (14.1 ± 9.7) cm^–1^. The reported IR spectra of MVKO and IMVKO in this work have removed the contributions of IMVKO and MVKO, respectively, from the observed spectra at low and high pressures, thereby correcting the previously reported spectra.^29^

**Table 1:** Comparison of Observed Vibrational Wavenumbers (in cm^–1^) and IR Intensities of Features in Group C with Those of the Convoluted Spectrum of C_2_H_3_C­(CH_3_)­IOO

mode	experiment		C_2_H_3_C(CH_3_)IOO^a^		mode descriptions^d^
	ν/cm^–1^	intensity^b^	ν/cm^–1^	intensity^c^	
ν_10_	1435	24	1421	14.9	C^(1)^H_2_ scissor
ν_11_	1371	51	1376	13.9	C^(4)^H_3_ umbrella
ν_12_	^e^		1305	0.1	HC^(2)^C^(1)^ bend
ν_13_	^e^		1247	7.0	C^(2)^C^(3)^ str. (conformers 1 and 3)
ν_13_	^e^		1205	4.4	C^(2)^C^(3)^ str. (conformer 2)
ν_14_	^e^		1170	4.4	OO str. (conformer 2)
ν_14_	^e^		1155	1.9	OO str. (conformer 3)
ν_14_	^e^		1145	1.7	OO str. (conformer 1)
ν_15_	1105	35	1108	24.0	CH *ip* bend/C^(4)^H_2_ wag
ν_16_	1060	100	1054	60.6	C^(3)^C^(4)^ str./C^(4)^H_2_ wag
ν_17_	986	49	1018	7.2	C^(2)^H *oop* bend (conformers 2 and 3)
ν_17_			1008	7.9	C^(2)^H *oop* bend (conformer 1)
ν_18_	964	16	985	2.7	CH *ip* bend/C^(4)^H_2_ wag
ν_19_	931	38	954	38.5	C^(1)^H_2_ wag
ν_20_	^e^		870	2.3	C^(2)^C^(3)^ str. (conformer 2)
ν_20_	^f^		848	7.8	C^(2)^C^(3)^ str. (conformers 1 and 3)

a Harmonic vibrational wavenumbers *x* of C_2_H_3_C­(CH_3_)­IOO-1, C_2_H_3_C­(CH_3_)­IOO-2, and C_2_H_3_C­(CH_3_)­IOO-3, scaled according to 0.9708 *x* + 9.3, and convoluted using their predicted Boltzmann population distribution of 40%, 31%, and 29%, respectively.

b Percentage IR intensities relative to the most intense band near 1060 cm^–1^.

c In units of km mol^–1^.

d Approximate mode description. str.: stretch; def.: deform; *ip*: in-plane; *oop*: out-of-plane. The carbon-atom numbering follows ref 29.

e Unidentified due to small intensity.

f Unidentified due to unsatisfactory signal-to-noise ratio caused by filter cutoff.

To rule out other species, we considered the second iodoperoxy adduct isomer, C­(CH_3_)­ICHCH_2_OO, produced via the addition of O_2_ to the terminal methylene moiety. This isomer has six conformers, with the three lowest-energy structures differing in energy by less than 3 kJ mol^–1^.^29^ The IR stick spectra of these conformers, using scaled harmonic vibrational wavenumbers and IR intensities (Table S3), are depicted in Figure S3b–d, and the Boltzmann-weighted convoluted spectrum (conformer 1:2:3 = 0.48:0.36:0.16), with fwhm of 4 cm^–1^ is shown in Table S4 and Figure S3e, and reproduced in Figure 3c. The four most intense bands were predicted near 1281/1272, 1137, 1113, and 950/936 cm^–1^, which deviate from observed bands by −90, 32, 6, and 14 cm^–1^, respectively. The observed features in group C are hence poorly matched to the predicted spectrum of C­(CH_3_)­ICHCH_2_OO, further confirming the identity of the observed adduct as IMVKO.

### 3.2 Relative IR Intensity of MVKO and C_2_H_3_C­(CH_3_)­IOO

We recorded the IR spectra of MVKO and IMVKO in N_2_ (14.9–194 Torr) and O_2_ (31.7–190.2 Torr) at 298 K. As shown in Figures S4 and S5, increasing the total pressure leads to a systematic increase in the intensities of the IMVKO adduct bands, accompanied by a corresponding decrease in the MVKO features. To quantify these changes, we integrated the absorbance of the B_6_ and B_7_ bands of MVKO (900–962 cm^–1^, denoted *I*_MVKO_) and the C_4_ band of IMVKO (1035–1085 cm^–1^, denoted *I*_IMVKO_). In addition to MVKO and IMVK, only MVK product bands were observed in the 850–1450 cm^–1^ region. Precise quantification required accounting for spectral overlaps from methyl vinyl ketone (MVK) and IMVKO in the 900–962 cm^–1^ region. First, the small MVK contribution was removed by monitoring its interference-free band (1220–1270 cm^–1^). Using an observed intensity ratio of 0.815 for the integrated absorbance in the 900–962 cm^–1^ region to that in the 1220–1270 cm^–1^ region, we subtracted this contribution from the integrated absorbance in the 900–962 cm^–1^ region. Typically, this subtraction is less than 5%. Second, the small IMVKO contribution to the same region was eliminated by scaling the integrated absorbance of its C_4_ band by a factor of 0.4 (the observed C_7_/C_4_ intensity ratio of 38/100). Following these corrections, the residual absorbance in the 900–962 cm^–1^ range was assigned exclusively to syn-MVKO.

Regarding conformational distribution, quantum-chemical calculations indicate that syn*-*cis-MVKO lies 6.1–7.4 kJ mol^–1^ above the syn*-*trans global minimum.^25,26^ Given the relatively low interconversion barrier, the Boltzmann distribution at 298 K predicts an equilibrium population of less than 8% for the syn-cis conformer. Furthermore, the predicted IR intensity for the O–O stretching (ν_15_) mode of syn*-cis*-MVKO is only 67% that of the syn-trans species. Due to its low population and weaker IR signature in the 900–962 cm^–1^ region, we focus our analysis on *syn-trans*-MVKO (hereafter referred to as syn-MVKO); the error introduced by neglecting syn-*cis*-MVKO is expected to be less than 5%. No spectral evidence for anti-MVKO was observed, consistent with its rapid unimolecular decay at room temperature. For simplicity, if there is no confusion, we denote syn-MVKO as MVKO, unless noted.

The temporal profiles of the integrated absorbance of syn-MVKO in N_2_ and O_2_ are shown in Figures S6 and S7, respectively. The rising signal corresponds to the formation reaction ^•^C_2_H_3_C­(CH_3_)I + O_2_, while the slow decay is attributed to secondary reactions, such as the self-reaction of syn-MVKO and its reaction with I atoms. To determine the initial integrated absorbance of MVKO, *I*_syn_ *_‑_*_MVKO,0_, we fitted the temporal profiles using a biexponential consecutive-reaction model

3 $$I_{sy\mathrm{n‐}MVKO}\left(t\right)=I_{sy\mathrm{n‐}MVKO,0}\times \frac{k_{1}}{k_{1}-k_{d}}\left[e^{{-k}_{d}t}-e^{{-k}_{1}t}\right]$$

in which *I*_syn‑MVKO_ is the integrated absorbance of syn-MVKO, *k*_1_ is the formation rate coefficient, *k*_d_ is the overall decay rate coefficient, and *t* is the reaction period. The fitted results are shown as solid red lines in Figures S6 and S7 for experiments in N_2_ and O_2_, respectively; derived *I*_syn‑MVKO,0_ are listed in Table S5.

Alternatively, assuming that the self-reaction of MVKO dominates the decay process, we fitted the decay temporal profiles with a second-order reaction model

4 $$1/I_{sy\mathrm{n‐}MVKO}\left(t\right)=1/I_{\mathrm{syn‐}MVKO,0}^{\prime }+2k_{self}t$$

in which *I*_syn‑MVKO,0_^′^ is the initial integrated absorbance of MVKO derived from this method, *k*_self_ is the self-reaction rate coefficient, and *t* is the reaction period. The fitted results are shown in Figures S8 and S9 for experiments in N_2_ and O_2_, respectively. The values derived from both models (Table S5) show satisfactory agreement, with an average absolute deviation of only 5.4% and a maximal deviation of 8%. We used *I*_syn‑MVKO,0_ from the biexponential fit for subsequent calculations because of its smaller statistical uncertainty.

Because the decay of the IMVKO adduct is significantly slower, its initial absorbance *I*_IMVKO,0_ was evaluated by integrating the 1035–1085 cm^–1^ region over the 10–20 μs interval. To convert the ratio *I*_IMVKO,0_/*I*_syn*‑*MVKO,0_ into the concentration ratio [IMVKO]_0_/[syn-MVKO]_0_, we need to determine the relative absorption cross-section ε_IMVKO_/ε_syn–MVKO_. If we follow the analysis of CH_2_OO/CH_2_IOO^51^ and assume that the sum of the initial concentrations [syn-MVKO]_0_ + [IMVKO]_0_ remains constant for a fixed initial precursor concentration, the relative cross-section ε_IMVKO_/ε_syn–MVKO_ can be derived from the slope of a plot of *I*_IMVKO,0_ vs *I*_syn*‑*MVKO,0_, as shown in Figure S10. Data from experiments in N_2_ and O_2_ (with different initial precursor concentrations in each set) are presented in green circles and blue squares, respectively. A simultaneous fit of the N_2_ and O_2_ data sets yielded a shared slope of −(0.36 ± 0.03), shown as solid red lines; the listed error represents one standard deviation in fitting. Individual fits of data from experiments in N_2_ and O_2_ yielded slopes of −(0.33 ± 0.03) and −(0.41 ± 0.05), respectively, with error limits that overlapped.

To validate this result, we compared it with quantum-chemical predictions at the B3LYP/aug-cc-pVTZ level. The IR intensity of the B_6_ and B_7_ bands of syn-*trans*-MVKO was predicted to be 171 km mol^–1^ (summing 127.4 km mol^–1^ for ν_15_ and 43.6 km mol^–1^ for ν_25_).^29^ For IMVKO, the weighted-average intensity of the C_4_ band across the three lowest-energy conformers was calculated to be 60.6 km mol^–1^, using predicted IR intensities of 56.8, 74.7, and 50.9 km mol^–1^ (ν_16_) of three lowest-energy conformers of IMVKO, with a Boltzmann distribution of 40:31:29. The resulting theoretical ratio of 0.35 (= 60.6/171) is in remarkable agreement with our experimental value of 0.36 ± 0.03, providing support to our quantitative yield determinations.

### 3.3 Relative Yields of MVKO with Respect to C_2_H_3_C­(CH_3_)­IOO

Under the assumptions that the initial species are limited to [syn-MVKO]_0_ + [IMVKO]_0_ = constant and only syn-MVKO was probed by IR absorption in the range 900–962 cm^–1^, the relative yield of syn-MVKO with respect to the combined observed concentrations, *y*_α_^rel^, can be expressed as

5 $$y_{\alpha }^{rel}=\frac{{\left[sy\mathrm{n‐}MVKO\right]}_{0}}{{\left[sy\mathrm{n‐}MVKO\right]}_{0}+{\left[IMVKO\right]}_{0}}$$

Rearranging this into a linear form gives

$$1/y_{\alpha }^{rel}=1+\frac{{\left[IMVKO\right]}_{0}}{{\left[sy\mathrm{n‐}MVKO\right]}_{0}}=1+\left(\frac{I_{IMVKO,0}}{I_{sy\mathrm{n‐}MVKO,0}}\right)\times \left(\frac{\varepsilon_{sy\mathrm{n‐}MVKO}}{\varepsilon_{IMVKO}}\right)$$ 6

Values of *y*_α_^rel^, derived from our IR data, are listed as a function of pressure in Table S6. In Figure 4, the plot of 1/*y*_α_^rel^ versus the total number of density [M] exhibits a clear linear pressure dependence (dashed lines). This behavior is consistent with a competitive mechanism where *y*_γ_ (the yield of the stabilized adduct IMVKO) increases with pressure

$$1/y_{\alpha }^{rel}=1+\left(y_{\gamma }/y_{\alpha }\right)=1+\left(k_{\gamma }/k_{\alpha }\right)\left[M\right]$$ 7

Linear least-squares fits to the data sets for N_2_ and O_2_ buffer gases yield

$$1/y_{\alpha }^{rel}=(1.13\pm 0.14)+{\left(1.10\pm 0.05\right)\times 10}^{-18}\left[N_{2}\right]$$ 8

$$1/y_{\alpha }^{rel}=(0.85\pm 0.05)+{\left(0.96\pm 0.01\right)\times 10}^{-18}\left[O_{2}\right]$$ 9

in which [N_2_] and [O_2_] are in molecules cm^–3^. Given the insignificant difference between the two buffer gases, a combined fit yields

$$1/y_{\alpha }^{rel}=(1.08\pm 0.11)+{\left(1.08\pm 0.04\right)\times 10}^{-18}\left[M\right]$$ 10

for MVKO formation, shown as a red dashed line in Figure 4. Our direct IR measurements are in excellent agreement with the UV-based observations of Lin et al.,^33^ who monitored the delayed secondary formation of syn-MVKO from IMVKO dissociation (Reaction 2), as represented by brown open triangles in Figure 4.

**Figure 4:**
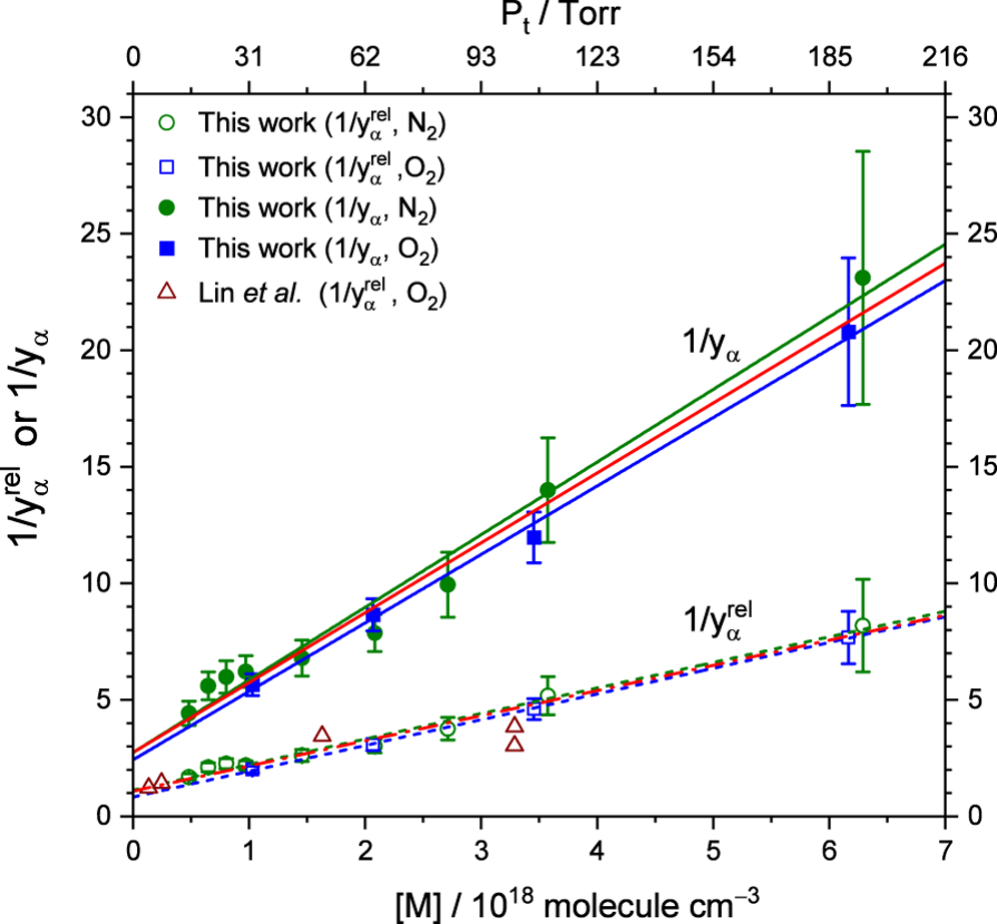
Inverse yields of *y*_α_^rel^ and *y*_α_ of syn-MVKO from the reaction C_2_H_3_C­(CH_3_)I + O_2_ as a function of total density [M]. *y*_α_^rel^ represents the relative yield of syn-MVKO, defined as [syn-MVKO]_0_/([syn-MVKO]_0_ + [IMVKO]_0_). Green open circles and blue open squares represent 1/*y*_α_^rel^ in experiments conducted in N_2_ and O_2_, respectively. The fitted equations are 1.13 + 1.10 × 10^–18^ [N_2_] (green dashed line) and 0.85 + 0.96 × 10^–18^ [O_2_] (blue dashed line), and 1.08 + 1.08 × 10^–18^ [M] (red dashed line, the combined fitting). Results from Lin et al.^33^ are shown as brown open triangles. *y*_α_ represents the absolute yield of syn-MVKO, defined as *y*_α_ = [syn-MVKO]_0_/[^•^C_2_H_3_C­(CH_3_)­I]_0_. Green solid circles and blue solid squares represent 1/*y*_α_ in experiments conducted in N_2_ and O_2_, respectively. The fitted equations are 2.74 + 3.12 × 10^–18^ [N_2_] (green solid line), 2.43 + 2.94 × 10^–18^ [O_2_] (blue solid line), and 2.74 + 3.00 × 10^–18^ [M] (red solid line, the combined fitting).

It should be noted that the present analysis was based on the assumption that reactions (1b) and (1d) are negligible, so that [syn-MVKO]_0_ + [IMVKO]_0_ = constant. If anti-MVKO is not negligible, following the discussion in Note SA of the Supporting Information, the relative yield derived from the above analysis should be

$$y_{\alpha +\beta }^{rel}=\frac{{\left[sy\mathrm{n‐}MVKO\right]}_{0}+{\left[ant\mathrm{i‐}MVKO\right]}_{0}}{{\left[sy\mathrm{n‐}MVKO\right]}_{0}+{\left[ant\mathrm{i‐}MVKO\right]}_{0}+{\left[IMVKO\right]}_{0}}$$ 11

rather than *y*_α_^rel^. While neither the IR nor the UV spectrum of anti-MVKO was explicitly detected in the reaction of ^•^C_2_H_3_C­(CH_3_)I + O_2_, likely due to its rapid unimolecular decay, any minor contribution it might have made to the B_6_ and B_7_ absorption band would not significantly alter our determination of *y*_α_^rel^.

The slope derived from Figure 4 (1/*y*_α_^rel^ vs [M]) is more than ten times that observed for the relative yield of the simplest Criegee intermediate, CH_2_OO. Ting et al. reported a slope of (9.1 ± 0.3) × 10^–20^ cm^3^ molecule^–1^ at 295 K,^52^ while Huang et al. found a similar value of (9.7 ± 0.3) × 10^–20^ cm^3^ molecule^–1^ at 343 K.^51^ These comparisons indicate that the IMVKO adduct is stabilized much more efficiently than CH_2_IOO as pressure increases.

According to Lin et al., based on the CCSD­(T)-F12b//B2PLYP calculations, the reaction of ^•^CH_2_I + O_2_ to form CH_2_IOO is exothermic by ∼110 kJ mol^–1^, whereas the formation of CH_2_OO + I is endothermic by ∼4 kJ mol^–1^.^37^ The reactions of ^•^C_2_H_3_C­(CH_3_)I + O_2_ to form IMVKO and syn-MVKO + I are exothermic by ∼65 and 6 kJ mol^–1^, respectively. The required energy for CH_2_IOO to dissociate into CH_2_OO + I, 114 kJ mol^–1^, is much larger than the corresponding value ∼59 kJ mol^–1^ for IMVKO → MVKO + I. The observed enhanced efficiency in stabilizing the IMVKO adduct likely stems from the significantly greater number of vibrational degrees of freedom and the presence of a methyl rotor in IMVKO, which facilitate rapid redistribution and dissipation of the internal energy of the nascent adduct upon collision with buffer-gas molecules.

As a consequence, IMVKO is produced efficiently even at low pressures. At 35 Torr (the conditions utilized in the previous study by Chung and Lee^29^), eq 10 predicts *y*_α_^rel^ ≈ 0.45, implying a ratio [syn-MVKO]: [IMVKO] = 0.45:0.55. This suggests that the previously reported syn-MVKO spectrum contained a substantial contribution from IMVKO, particularly the most intense band near 1060 cm^–1^. The absence of this feature in our purified syn-MVKO spectrum (Figure 2c) confirms that the present results provide a more accurate representation of the pure syn-*trans*-MVKO infrared signature.

### 3.4 Estimate of Absolute Yield of MVKO

Lin et al. reported a notably small combined yield of syn-MVKO and IMVKO, *y*_α_ + *y*_γ_ = (0.22 ± 0.10) at 299 K over the pressure range 30–700 Torr.^37^ To verify this small yield, we further estimated the absolute yield of syn-MVKO, *y*_α_, by relating the initial concentrations

yα=[syn‐MVKO]0[C2H3C(CH3)I•]0 12

The initial concentration of syn-MVKO, [syn-MVKO]_0_, was derived from the extrapolated integrated absorbance at time zero (*I*_syn__‑__MVKO,0_) using IR intensities (ν_15_ and ν_25_) calculated at the B3LYP/aug-cc-pVTZ level. The initial radical-precursor concentration, [^•^C_2_H_3_C­(CH_3_)­I]_0_, was assumed to be equal to the consumption of the precursor (*Z*)-(CH_2_I)­HC=C­(CH_3_)­I, [^•^C_2_H_3_C­(CH_3_)­I]_0_ = −Δ­[(CH_2_I)­HC=C­(CH_3_)­I]_0_, determined by monitoring band depletion in the 1120–1210 and 1265–1320 cm^–1^ regions (which are free from product interference). The experimental determination of absorption cross sections for these precursor bands is detailed in Note SB of the Supporting Information and Table S7. It should be noted that the use of quantum-chemically predicted IR intensities of syn-*trans*-MVKO introduces systematic uncertainty. Based on the average experimental-to-predicted intensity ratio for the precursor in Table S7, (0.66 ± 0.11), we estimate an uncertainty of ±35% for the predicted IR intensity, which, in extreme cases, could reach a factor of 2. Nevertheless, this calculation provides a valuable estimate of the absolute yield of syn-MVKO and its comparison to other Criegee systems.

The absolute yields of syn-MVKO (*y*_α_) derived in this study are summarized in Table S6. The inverse absolute yield, 1/*y*_α_, is plotted as a function of total density [M] (M = N_2_, O_2_) in Figure 4. Linear least-squares fits for each buffer gas yield

$${1/y}_{\alpha }=(2.74\pm 0.47)+{\left(3.12\pm 0.17\right)\times 10}^{-18}\left[N_{2}\right]$$ 13

$${1/y}_{\alpha }=(2.43\pm 0.48)+{\left(2.94\pm 0.13\right)\times 10}^{-18}\left[O_{2}\right]$$ 14

in which [N_2_] and [O_2_] are in unit of molecules cm^–3^. As these results show no significant dependence on the identity of the buffer gas, a combined fit provides the following relationship

$${1/y}_{\alpha }=(2.74\pm 0.40)+{\left(3.00\pm 0.13\right)\times 10}^{-18}\left[M\right]$$ 15

At 14.9 Torr, our experimental *y*_α_ value was 0.23 ± 0.03 (similar to 0.24 ± 0.02 from eq 15); the error limit does not account for the possible 35% error in IR intensity calculations. Using our relative yield 1/*y*_α_^rel^ = 1.69 ± 0.14, we derive a combined yield *y*_α_ + *y*_γ_ = 0.38 ± 0.06. At 194 Torr, where *y*_α_ drops to 0.04 ± 0.01 and 1/*y*_α_^rel^ = 8.19 ± 1.99, we find *y*_α_ + *y*_γ_ = 0.35 ± 0.12. Lin et al. previously estimated a pressure-independent total yield of (0.22 ± 0.10) at 299 K and 30–700 Torr for MVKO by assuming the eventual dissociation of all IMVKO into syn-MVKO.^37^ Our averaged total yield, *y*_α_ + *y*_γ_ = 0.37 ± 0.06, is approximately 1.7 times the value reported by Lin et al. While this factor is larger than the typical error expected from IR intensity calculations, the error limits of the two studies do overlap slightly. Furthermore, even with our estimate of a higher yield, more than 60% of the products from the ^•^C_2_H_3_C­(CH_3_)I + O_2_ reaction remain unaccounted for. This pressure-independent deficiency may be attributed to the anti-MVKO channel, which rapidly decomposes into secondary products, or other unknown channels. However, the potential pressure independence of the anti-MVKO yield remains an open question. These findings underscore the need for further experimental and theoretical investigations to fully decipher the complex, pressure-dependent branching ratios between syn-MVKO, anti-MVKO, the IMVKO adduct, and other channels from the reaction of ^•^C_2_H_3_C­(CH_3_)I + O_2_.

## 4 Conclusion

By careful comparison of the transient IR spectra recorded following photolysis at 248 nm of a flowing mixture of (*Z*)-(CH_2_I)­HC=C­(CH_3_)I and O_2_ at 14.9 and 179 Torr (298 K), we assigned seven bands observed at high pressure to the iodoperoxy adduct IMVKO, C_2_H_3_C­(CH_3_)­IOO, showing excellent agreement with the Boltzmann-weighted convoluted spectrum from the three lowest-energy conformers of IMVKO. A more accurate MVKO spectrum, correcting prior reports that contained significant contributions of IMVKO bands near 1060 cm^–1^, is also reported. By simultaneously monitoring IR bands of syn-MVKO and IMVKO, we determined the relative IR intensity of IMVKO (ν_16_) with respect to MVKO (ν_15_ and ν_25_) as 0.36 ± 0.03, consistent with the value of 0.35 predicted by the B3LYP/aug-cc-pVTZ method.

By performing experiments in the pressure range of 14.9–194 Torr, we quantified the pressure dependence of the yield of syn-MVKO relative to the total yield of syn-MVKO and IMVKO, *y*_α_^rel^, to follow 1/*y*_α_^rel^ = (1.08 ± 0.11) + (1.08 ± 0.04) × 10^–18^ [M]; the difference between O_2_ and N_2_ as a quencher is insignificant. The value 1.08 × 10^–18^ is more than ten times that reported for CH_2_OO, indicating enhanced efficiency in dissipation of the internal energy of IMVKO by buffer gases, likely stemming from the significantly greater number of vibrational degrees of freedom and the presence of a methyl rotor in IMVKO. Furthermore, incorporating quantum-chemically predicted IR intensities of MVKO and the experimentally determined IR intensity of the precursor, the absolute yield (*y*_α_) of MVKO was estimated to be 1/*y*_α_ = (2.74 ± 0.40) + (3.00 ± 0.13) × 10^–18^ [M], significantly larger than the previous report. Even with this larger yield, ∼(37 ± 6)% for syn-MVKO and IMVKO across 15–194 Torr, a significant amount of the products from the ^•^C_2_H_3_C­(CH_3_)I + O_2_ reaction remains unaccounted for.

## Supplementary Material



## Notes

Effect of anti-MVKO on measured relative yield *y*_α_^rel^ of syn-MVKO (Note SA); Measurements of IR intensities of 1,3-diiodo-but-2-ene (Note SB). Scale harmonic vibrational wavenumbers and IR intensities of nine conformers of C_2_H_3_C­(CH_3_)­IOO and six conformers of C­(CH_3_)­ICHCH_2_OO, convolution of calculated spectrum of three conformers C_2_H_3_C­(CH_3_)­IOO and C­(CH_3_)­ICHCH_2_OO, fitted values of *I*_syn*‑*MVKO,0_ and *I*_syn‑MVKO,0_^′^ at various pressures, summary of relative yields (*y*_α_^rel^) and estimated absolute yields (*y*_α_) at various pressures, Experimental conditions and measurements of IR intensities (in km mol^–1^) of 1,3-diiodo-but-2-ene (7 tables). Observed and processed spectra in the region 1450–850 cm^–1^ upon photolysis of a flowing mixture of (*Z*)-(CH_2_I)­HC=C­(CH_3_)­I/O_2_ at 194 Torr, convolution of spectra of three conformers of the adduct C_2_H_3_C­(CH_3_)­IOO and C­(CH_3_)­ICHCH_2_OO, processed spectra of (*Z*)-(CH_2_I)­HC=C­(CH_3_)I in N_2_ and O_2_ at various pressures, temporal profiles of *I*_syn_ *_‑_*_MVKO_ in N_2_ and O_2_ at various pressures, temporal profiles of 1/*I*_syn‑_ _MVKO_ in N_2_ and O_2_ at various pressures, plot of *I*_IMVKO,0_ versus *I*_syn*‑*MVKO,0_ at various pressures (10 figures) (PDF)
