# Supplementary material for: Infrared Spectra and Pressure-Dependent Yields of the Criegee Intermediate Methyl Vinyl Ketone Oxide and Its Iodoperoxy Adduct
Source: J Phys Chem A. 2026 Jun 1;130(23):4392–400. doi: 10.1021/acs.jpca.6c02328 (PMC13267084; doi:10.1021/acs.jpca.6c02328)
Supplement: Supplementary file 1 [file jp6c02328_si_001.pdf]

Supporting Information

Infrared Spectra and Pressure-Dependent Yields of the Criegee Intermediate Methyl Vinyl Ketone Oxide and Its Iodoperoxy Adduct

Ju-Yin Hsu,[a] and Yuan-Pern Lee*[ab]

[a]*Department of Applied Chemistry and Institute of Molecular Science, National Yang Ming Chiao Tung University, 1001, Ta-Hsueh Road, Hsinchu 300093, Taiwan.*

[b]*Center for Emergent Functional Matter Science, National Yang Ming Chiao Tung University, Hsinchu 300093, Taiwan*

Email: yplee@nycu.edu.tw

Table of Contents

Note SA. Effect of *anti*-MVKO on measured relative yield y_α^{rel} of *syn*-MVKO.................S1

Note SB. Measurements of IR intensities of 1,3-diiodo-but-2-ene S2

Table S1. Scale harmonic vibrational wavenumbers (cm^{-1}) and IR intensities ($km\ mol^{-1}$) of nine conformers of $C_2H_3C(CH_3)IOO$ predicted with the B3LYP/aug-cc-pVTZ-pp method..S4

Table S2. Comparison of observed vibrational wavenumbers (in cm^{-1}) and relative IR intensities of features in group C with the scaled harmonic vibrational wavenumbers and IR intensities of three conformers $C_2H_3C(CH_3)IOO$ and the convoluted values.........S7

Table S3. Scale harmonic vibrational wavenumbers (cm^{-1}) and IR intensities ($km\ mol^{-1}$) of six conformers of $C(CH_3)ICHCH_2OO$ predicted with the B3LYP/aug-cc-pVTZ-pp method..S8

Table S4. Convolution of the scaled harmonic vibrational wavenumbers (in cm^{-1}) and IR intensities (in $km\ mol^{-1}$) of three lowest-energy conformers $C(CH_3)ICHCH_2OO$...........S10

Table S5. Fitted values of $I_{syn\text{-MVKO},0}$ and $I'_{syn-\mathrm{MVKO},0}$ in N_2 and O_2 at various pressures ..S11

Table S6. Summary of relative yields (y_α^{rel}) and estimated absolute yields (y_α) at various pressures..S12

Table S7. Experimental conditions and measurements of IR intensities (in $km\ mol^{-1}$) of 1,3-diiodo-but-2-ene...S13

Figure S1. Observed and processed spectra in the region 1450−850 cm^{-1} upon photolysis at 248 nm of a flowing mixture of (*Z*)-$(CH_2I)HC{=}C(CH_3)I/O_2$ (0.05/15/179 Torr) S14

Figure S2. Convolution of spectra of three conformers of the adduct $C_2H_3C(CH_3)IOO$ S15

Figure S3. Convolution of three conformers of the adduct $C(CH_3)ICHCH_2OO$.................S16

Figure S4. Processed spectra in the region 850–1450 cm^{-1} recorded 10–20 μs after photolysis at 248 nm of a flowing mixture of (*Z*)-$(CH_2I)HC{=}C(CH_3)I/O_2/N_2$ at various pressures..S17

Figure S5. Processed spectra in the region 850–1450 cm^{-1} recorded 10–20 μs after photolysis at 248 nm of a flowing mixture of (*Z*)-$(CH_2I)HC{=}C(CH_3)I/O_2$ at various pressures..S18

Figure S6. Temporal profiles of $I_{syn\text{-MVKO}}$ in N_2 at various pressures S19

Figure S7. Temporal profiles of $I_{syn\text{-MVKO}}$ in O_2 at various pressures..................................S20

Figure S8. Temporal profiles of $1/I_{syn\text{-MVKO}}$ in N_2 at various pressures...............................S21

Figure S9. Temporal profiles of $1/I_{syn\text{-MVKO}}$ in O_2 at various pressures...............................S22

Figure S10. Plot of integrated absorbance of $C_2H_3C(CH_3)IOO$ ($I_{IMVKO,0}$) versus that of $C_2H_3C(CH_3)OO$ ($I_{syn\text{-}MVKO,0}$) at various pressures in two sets of experiments S23
References S24

Note SA. Effect of *anti*-MVKO on measured relative yield y_{α}^{rel} of *syn*-MVKO

If some *anti*-MVKO were present and were also probed within the spectral region of integration, we defined the relative combined yield of *syn*-MVKO + *anti*-MVKO, $y_{\alpha+\beta}^{\text{rel}}$, as

$$y_{\alpha+\beta}^{\text{rel}} = \frac{[syn\text{–MVKO}]_0 + [anti\text{–MVKO}]_0}{[syn\text{–MVKO}]_0 + [anti\text{–MVKO}]_0 + [\text{IMVKO}]_0} \quad \text{(S1)}$$

Accordingly,

$$[syn\text{-MVKO}]_0 + [anti\text{-MVKO}]_0 + [\text{IMVKO}]_0 = \text{constant} \quad \text{(S2)}$$

Assume that

$$[anti\text{-MVKO}]_0 / [syn\text{-MVKO}]_0 = y_\beta / y_\alpha = m, \quad \text{(S3)}$$

we derive

$$[syn\text{-MVKO}]_0 + [anti\text{-MVKO}]_0 = (1 + m)\,[syn\text{-MVKO}]_0 \quad \text{(S4)}$$

Under the assumption that $(1 + m)\times[syn\text{-MVKO}]_0 + [\text{IMVKO}]_0$ remains constant, then

$$(1 + m) \times \left(\frac{I_{syn\text{–MVKO},0}}{\varepsilon_{syn\text{–MVKO}}}\right) = \text{constant} - \left(\frac{I_{\text{IMVKO},0}}{\varepsilon_{\text{IMVKO}}}\right) \quad \text{(S5)}$$

in which I represents the integrated absorbance and ε represents the IR cross-section in the integrated region. However, if *anti*-MVKO were present in the integrated area in the experiment, the observed integrated absorbance is $I_{\text{MVKO},0}'$ rather than $I_{\text{syn-MVKO},0}$,

$$I_{\text{MVKO},0}' = l\,([syn\text{-MVKO}]_0\,\varepsilon_{\text{syn-MVKO}} + [anti\text{-MVKO}]_0\,\varepsilon_{\text{anti-MVKO}})$$

$$= l\,[syn\text{-MVKO}]_0\,(\varepsilon_{\text{syn-MVKO}} + m\,\varepsilon_{\text{anti-MVKO}}) \quad \text{(S6)}$$

$$I_{\text{syn-MVKO},0} = l\,[syn\text{-MVKO}]_0\,\varepsilon_{\text{syn-MVKO}} \quad \text{(S7)}$$

in which l is the absorption length. Consequently, equation (S5) becomes

$$(1 + m) \times \left(\frac{I_{\text{MVKO},0}'}{\varepsilon_{syn\text{–MVKO}}}\right)\left(\frac{I_{syn\text{–MVKO},0}}{I_{\text{MVKO},0}'}\right) = \text{constant} - \left(\frac{I_{\text{IMVKO},0}}{\varepsilon_{\text{IMVKO}}}\right) \quad \text{(S8)}$$

Substitution equations (S6) and (S7) into (S8),

$$(1 + m) \times \left(\frac{I_{\text{MVKO},0}'}{\varepsilon_{syn\text{–MVKO}}}\right)\left(\frac{\varepsilon_{syn\text{–MVKO}}}{\varepsilon_{syn\text{–MVKO}} + m\,\varepsilon_{anti\text{–MVKO}}}\right) = \text{constant} - \left(\frac{I_{\text{IMVKO},0}}{\varepsilon_{\text{IMVKO}}}\right) \quad \text{(S9)}$$

A linear fit of $I_{\text{IMVKO},0}$ versus $I_{\text{MVKO},0}'$ obtained from experiments with constant initial $[C_2H_3C(CH_3)I]_0$ yields a slope (S) :

$$S = (1 + m) \times \left(\frac{\varepsilon_{\text{IMVKO}}}{\varepsilon_{syn\text{–MVKO}} + m\,\varepsilon_{anti\text{–MVKO}}}\right) \quad \text{(S10)}$$

Rearranging Equation (S1) gives

$$1/y_{\alpha+\beta}^{\text{rel}} = 1 + \frac{[\text{IMVKO}]_0}{[syn\text{–MVKO}]_0 + [anti\text{–MVKO}]_0} = 1 + \frac{[\text{IMVKO}]_0}{(1 + m)\,[syn\text{–MVKO}]_0}$$

$$= 1 + \left(\frac{1}{1 + m}\right) \times \left(\frac{I_{\text{IMVKO},0}}{I_{syn\text{–MVKO},0}}\right) \times \left(\frac{\varepsilon_{syn\text{–MVKO}}}{\varepsilon_{\text{IMVKO}}}\right)$$

$$= 1 + \left(\frac{1}{1+m}\right) \times \left(\frac{I_{\mathrm{IMVKO,0}}}{I_{\mathrm{MVKO,0}}{}'}\right)\left(\frac{I_{\mathrm{MVKO,0}}{}'}{I_{\mathrm{syn-MVKO,0}}}\right) \times \left(\frac{\varepsilon_{syn-\mathrm{MVKO}}}{\varepsilon_{\mathrm{IMVKO}}}\right)$$

$$= 1 + \left(\frac{1}{1+m}\right) \times \left(\frac{I_{\mathrm{IMVKO,0}}}{I_{\mathrm{MVKO,0}}{}'}\right)\left(\frac{\varepsilon_{syn-\mathrm{MVKO}} + m\,\varepsilon_{anti-\mathrm{MVKO}}}{\varepsilon_{syn-\mathrm{MVKO}}}\right) \times \left(\frac{\varepsilon_{syn-\mathrm{MVKO}}}{\varepsilon_{\mathrm{IMVKO}}}\right) \quad \text{(S11)}$$

Substituting S into $1/y^{\mathrm{rel}}_{\alpha+\beta}$ gives

$$1/y^{\mathrm{rel}}_{\alpha+\beta} = 1 + \left(\frac{I_{\mathrm{IMVKO,0}}}{I_{\mathrm{MVKO,0}}{}'}\right)/S \quad \text{(S12)}$$

That means, the relative yield $1/y^{\mathrm{rel}}_{\alpha}$ obtained using our method (discussed in the main text) should be regarded as $1/y^{\mathrm{rel}}_{\alpha+\beta}$.

If only the yields of *syn*-MVKO and IMVKO is taken into account,

$$1/y^{\mathrm{rel}}_{\alpha} = 1 + \frac{[I\mathrm{MVKO}]_0}{[syn-\mathrm{MVKO}]_0} = 1 + \left(\frac{I_{\mathrm{IMVKO,0}}}{I_{\mathrm{syn-MVKO,0}}}\right) \times \left(\frac{\varepsilon_{syn-\mathrm{MVKO}}}{\varepsilon_{IMVKO}}\right)$$

$$= 1 + \left(\frac{I_{\mathrm{IMVKO,0}}}{I_{\mathrm{MVKO,0}}{}'}\right)\left(\frac{I_{\mathrm{MVKO,0}}{}'}{I_{\mathrm{syn-MVKO,0}}}\right) \times \left(\frac{\varepsilon_{syn-\mathrm{MVKO}}}{\varepsilon_{IMVKO}}\right)$$

$$= 1 + \left(\frac{I_{\mathrm{IMVKO,0}}}{I_{\mathrm{MVKO,0}}{}'}\right)\left(\frac{\varepsilon_{syn-\mathrm{MVKO}} + m\,\varepsilon_{anti-\mathrm{MVKO}}}{\varepsilon_{syn-\mathrm{MVKO}}}\right) \times \left(\frac{\varepsilon_{syn-\mathrm{MVKO}}}{\varepsilon_{IMVKO}}\right)$$

$$= 1 + \left(\frac{I_{\mathrm{IMVKO,0}}}{I_{\mathrm{MVKO,0}}{}'}\right)\left(\frac{\varepsilon_{syn-\mathrm{MVKO}} + m\,\varepsilon_{anti-\mathrm{MVKO}}}{\varepsilon_{IMVKO}}\right)$$

$$= 1 + \left(\frac{I_{\mathrm{IMVKO,0}}}{I_{\mathrm{MVKO,0}}{}'}\right)\ (1+m)/\mathrm{S} \quad \text{(S13)}$$

When $m\,\varepsilon_{anti-\mathrm{MVKO}} \approx 0$, that is, either negligible *anti*-MVKO was present ($m \approx 0$) or $\varepsilon_{anti-\mathrm{MVKO}}$ is very small in the integrated region, equation (S13) is equivalent to equation (6) in the main text.

Note SB. Measurements of IR intensities of 1,3-diiodo-but-2-ene

A method similar to that reported by Lin et al.[1] was employed to determine the absolute IR absorption cross sections of the precursor 1,3-diiodo-but-2-ene. The vapor of 1,3-diiodo-but-2-ene was admitted to the reaction chamber using N_2 as a carrier gas. By maintaining a constant flow rate of the carrier gas (and hence a constant absorbance) for an extended period, we measure the precursor's weight loss. According to Beer's law :

$$\int A\,d\tilde{\nu} = \int \varepsilon\,d\tilde{\nu} \times \frac{1000}{N_A} \times b \times c \times \log(e)$$

$$= \int \varepsilon\,d\tilde{\nu} \times \frac{1000}{N_A} \times b \times \frac{n}{V} \times 0.4343 \quad \text{(S14)}$$

in which $\int A\,d\tilde{\nu}$ is the integrated absorbance (cm^{-1}) of a given vibrational mode of the precursor, $\int \varepsilon\,d\tilde{\nu}$ is the corresponding IR absorption intensity (km mol^{-1}), b is the effective IR path length (cm), c is the concentration (molecule cm^{-3}), n is the number of molecules, and V is the volume (cm^3). The number of molecules n and the volume V are expressed as :

$$n = \frac{\Delta W}{M} \times N_A \tag{S15}$$

$$V = F \times t \tag{S16}$$

in which ΔW is the mass (g) of precursor consumed, M is the molecular weight (g mol^{-1}) of 1,3-diiodo-but-2-ene, N_A is the Avogadro number, F is the flow rate (cm^3 s^{-1}) of the N_2 carrier gas, and t (s) is the duration of the experiment. The flow rate (in STP sccm) was converted to the actual flow rate (in cm^3 s^{-1}) by the following equation :

$$F\ (\text{STP sccm}) = F\ (\text{cm}^3\ \text{s}^{-1}) \times \frac{760}{P_\text{T}} \times \frac{298}{273} \times \frac{1}{60} \tag{S17}$$

By measuring F, t and ΔW, the IR cross sections of each vibrational mode can be derived. A total of six experiments performed under different pressures and durations were analyzed. The experimental parameters and the derived IR absorption cross sections for each vibrational mode are summarized in Table S7.

Table S1. Scale Harmonic Vibrational Wavenumbers (cm^{-1}) and IR Intensities (km mol^{-1}) of Nine Conformers of $C_2H_3C(CH_3)IOO$ Predicted with the B3LYP/aug-cc-pVTZ-pp Method

mode	$C_2H_3C(CH_3)IOO$-1		$C_2H_3C(CH_3)IOO$-2		$C_2H_3C(CH_3)IOO$-3	
	ν^a/cm^{-1}	intensity	ν^a/cm^{-1}	intensity	ν^a/cm^{-1}	intensity
ν_1	3148	3.7	3158	1.8	3146	3.5
ν_2	3083	0.3	3085	1.0	3102	1.5
ν_3	3073	0.5	3074	0.1	3068	3.0
ν_4	3069	2.2	3072	3.2	3062	1.8
ν_5	3041	4.5	3035	4.4	3037	4.2
ν_6	2961	9.8	2957	9.1	2964	8.9
ν_7	1654	1.0	1648	1.2	1649	1.1
ν_8	1457	4.3	1456	4.9	1456	2.2
ν_9	1452	2.7	1443	2.1	1454	5.5
ν_{10}	1421	15.7	1423	13.2	1420	15.8
ν_{11}	1376	15.4	1377	13.6	1383	12.2
ν_{12}	1305	0.1	1305	0.1	1299	0.7
ν_{13}	1247	9.3	1206	14.3	1245	11.2
ν_{14}	1145	4.2	1171	14.0	1155	6.4
ν_{15}	1108	31.7	1104	10.2	1102	28.2
ν_{16}	1054	56.8	1051	74.7	1054	50.9
ν_{17}	1008	13.4	1021	10.0	1017	14.1
ν_{18}	985	3.6	1003	8.2	985	4.1
ν_{19}	954	39.3	957	37.3	953	38.6
ν_{20}	847	11.4	871	7.3	851	11.0
ν_{21}	767	69.8	755	61.0	735	75.2
ν_{22}	680	11.9	687	26.3	701	6.1
ν_{23}	569	4.8	570	6.8	594	9.7
ν_{24}	484	27.7	523	19.6	462	20.1
ν_{25}	416	8.3	414	10.9	423	8.2
ν_{26}	332	2.5	330	4.0	332	0.8
ν_{27}	286	0.6	269	0.3	268	2.3
ν_{28}	262	0.5	260	0.4	258	0.8
ν_{29}	249	4.9	245	6.9	246	6.4
ν_{30}	219	4.5	223	2.7	230	1.8
ν_{31}	213	1.0	208	0.3	215	1.8
ν_{32}	102	0.1	96	0.4	104	0.2
ν_{33}	89	0.2	71	0.1	96	0.1

mode	$C_2H_3C(CH_3)IOO$-4		$C_2H_3C(CH_3)IOO$-5		$C_2H_3C(CH_3)IOO$-6	
	ν^a/cm^{-1}	intensity	ν^a/cm^{-1}	intensity	ν^a/cm^{-1}	intensity
ν_1	3148	3.9	3154	2.0	3155	1.7
ν_2	3083	0.2	3079	0.9	3079	1.5
ν_3	3069	2.9	3069	1.9	3069	2.1
ν_4	3057	3.0	3058	3.3	3053	4.0
ν_5	3039	2.7	3032	4.6	3042	2.2
ν_6	2969	4.0	2960	9.1	2973	3.3
ν_7	1652	1.0	1648	0.9	1648	0.4
ν_8	1456	2.6	1453	6.1	1455	6.7
ν_9	1455	4.7	1447	2.8	1445	1.8
ν_{10}	1419	16.0	1423	14.4	1421	14.2
ν_{11}	1375	11.1	1381	10.9	1375	13.2
ν_{12}	1306	0.3	1303	0.1	1301	0.0
ν_{13}	1212	9.5	1199	14.5	1183	10.8
ν_{14}	1131	15.3	1137	10.5	1129	8.7
ν_{15}	1098	20.7	1104	13.9	1085	8.9
ν_{16}	1059	83.7	1061	79.9	1067	107.5
ν_{17}	1005	11.2	1019	7.8	1015	3.8
ν_{18}	984	4.5	995	6.8	999	9.4
ν_{19}	952	38.0	954	38.7	956	33.9
ν_{20}	881	18.1	872	7.6	885	9.6
ν_{21}	740	74.6	754	58.4	715	66.7
ν_{22}	712	1.4	681	22.4	711	17.0
ν_{23}	538	9.2	615	21.1	626	10.6
ν_{24}	522	9.2	514	6.9	491	3.3
ν_{25}	394	3.1	365	10.0	378	3.6
ν_{26}	324	4.4	357	1.2	341	3.5
ν_{27}	288	1.9	268	0.9	280	0.7
ν_{28}	260	1.4	251	2.3	253	1.5
ν_{29}	249	2.8	246	3.8	247	3.8
ν_{30}	223	2.3	235	0.9	222	2.2
ν_{31}	181	2.2	194	1.1	192	0.8
ν_{32}	98	0.2	132	0.1	106	0.2
ν_{33}	85	0.5	101	0.4	76	0.9

mode	$C_2H_3C(CH_3)IOO$-7		$C_2H_3C(CH_3)IOO$-8		$C_2H_3C(CH_3)IOO$-9	
	ν^a/cm^{-1}	intensity	ν^a/cm^{-1}	intensity	ν^a/cm^{-1}	intensity
ν_1	3147	3.5	3148	3.2	3145	3.7
ν_2	3067	1.8	3069	0.4	3065	1.9
ν_3	3061	3.1	3067	3.2	3053	4.1
ν_4	3045	3.3	3040	6.1	3051	0.3
ν_5	3029	5.9	3035	3.3	3036	3.8
ν_6	2958	11.0	2956	12.5	2969	4.3
ν_7	1669	6.9	1670	6.2	1665	7.9
ν_8	1452	4.7	1456	3.4	1455	4.5
ν_9	1447	1.8	1443	1.6	1445	2.1
ν_{10}	1416	21.2	1417	21.9	1416	17.5
ν_{11}	1380	9.8	1373	13.0	1373	10.7
ν_{12}	1301	4.0	1302	4.9	1292	4.9
ν_{13}	1192	21.5	1192	37.1	1167	53.7
ν_{14}	1146	15.4	1143	3.0	1129	4.6
ν_{15}	1083	44.9	1085	46.6	1090	56.3
ν_{16}	1060	30.8	1051	22.6	1040	14.7
ν_{17}	1024	6.6	1023	10.1	1019	8.1
ν_{18}	987	3.4	993	4.7	999	6.2
ν_{19}	962	36.9	970	38.5	963	36.0
ν_{20}	862	11.7	854	13.2	876	11.5
ν_{21}	749	42.2	756	33.7	725	27.3
ν_{22}	689	13.0	657	13.4	676	5.4
ν_{23}	568	14.9	595	11.9	632	21.3
ν_{24}	501	4.1	485	12.1	435	6.5
ν_{25}	419	14.9	397	11.7	377	2.8
ν_{26}	323	1.2	327	4.1	361	1.0
ν_{27}	285	3.5	316	0.4	307	4.4
ν_{28}	256	1.6	265	0.1	264	1.2
ν_{29}	255	1.5	260	2.2	251	0.1
ν_{30}	232	0.5	220	0.7	193	1.1
ν_{31}	178	0.6	186	0.4	187	0.4
ν_{32}	91	0.4	88	0.3	109	0.8
ν_{33}	72	0.4	66	0.3	80	0.3

[a]Harmonic vibrational wavenumber x scaled according to $0.9708\,x + 9.3$; see the main text.

Table S2. Comparison of Observed Vibrational Wavenumbers (in cm^{-1}) and Relative IR Intensities of Features in Group C with the Scaled Harmonic Vibrational Wavenumbers and IR Intensities of Three Conformers $C_2H_3C(CH_3)IOO$ and the Convoluted Values

mode	$C_2H_3C(CH_3)IOO$ conformers[a]			convoluted[b]	experiment
	$C_2H_3C(CH_3)IOO$-1	$C_2H_3C(CH_3)IOO$-2	$C_2H_3C(CH_3)IOO$-3		
ν_{10}	1421[c] (15.7)[d]	1422[c] (13.2)[d]	1420[c] (15.8)[d]	1421 (14.9)[d]	1435 (24)[e]
ν_{11}	1376 (15.4)	1376 (13.6)	1383 (12.2)	1376 (13.9)	1371 (51)
ν_{12}	1305 (0.1)	1304 (0.1)	1299 (0.7)	1305 (0.1)	[f]
ν_{13}	1247 (9.3)		1245 (11.2)	1247 (7.0)	[f]
ν_{13}		1205 (14.3)		1205 (4.4)	[f]
ν_{14}		1170 (14.0)		1170 (4.4)	[f]
ν_{14}			1155 (6.4)	1155 (1.9)	[f]
ν_{14}	1145 (4.2)			1145 (1.7)	[f]
ν_{15}	1108 (31.7)	1103 (10.2)	1102 (28.2)	1108 (24.0)	1105 (35)
ν_{16}	1054 (56.8)	1050 (74.7)	1054 (50.9)	1054 (60.6)	1060 (100)
ν_{17}		1020 (10.0)	1017 (14.1)	1018 (7.2)	986 (49)
ν_{17}	1008 (13.4)			1008 (7.9)	
ν_{18}	985 (3.6)	1002 (8.2)	985 (4.1)	985 (2.7)	964 (16)
ν_{19}	954 (39.3)	956 (37.3)	953 (38.6)	954 (38.5)	931 (38)
ν_{20}		870 (7.3)		870 (2.3)	[f]
ν_{20}	847(11.4)		851 (11.0)	848 (7.8)	[g]

[a]Predicted with the B3LYP/aug-cc-pVTZ-pp method. [b]Constructed from $C_2H_3C(CH_3)IOO$-1, $C_2H_3C(CH_3)IOO$-2, and $C_2H_3C(CH_3)IOO$-3 using their predicted Bolzmann population distribution of 40%, 31%, and 29%, respectively. [c]Harmonic vibrational wavenumber (cm^{-1}) scaled with $y = 0.9708\ x + 9.3$, in which x is the harmonic vibrational wavenumber. [d]IR intensities in km mol^{-1} are given in parentheses. [e]The percentage integrated IR intensities relative to the most intense band at 1060 cm^{-1} are listed in parentheses. [f]Unidentified due to small intensity. [g]Unidentified due to unsatisfactory signal-to-noise ratio caused by filter cutoff.

Table S3. Scale Harmonic Vibrational Wavenumbers (cm^{-1}) and IR Intensities (km mol^{-1}) of Six Conformers of $C(CH_3)ICHCH_2OO$ Predicted with the B3LYP/aug-cc-pVTZ-pp Method

mode	(*Z*)-$C(CH_3)ICHCH_2OO$-1		(*Z*)-$C(CH_3)ICHCH_2OO$-2		(*Z*)-$C(CH_3)ICHCH_2OO$-3	
	ν^a/cm^{-1}	intensity	ν^a/cm^{-1}	intensity	ν^a/cm^{-1}	intensity
ν_1	3075	2.0	3045	6.5	3046	4.5
ν_2	3005	1.4	3032	1.7	3033	2.5
ν_3	2993	12.1	3016	11.3	3015	11.7
ν_4	2992	5.0	3006	4.6	3005	5.0
ν_5	2956	8.6	2978	6.5	2973	11.5
ν_6	2880	13.0	2948	12.8	2948	13.8
ν_7	1609	24.7	1668	32.9	1669	23.6
ν_8	1423	4.1	1453	4.6	1453	4.4
ν_9	1410	4.8	1450	6.1	1439	5.4
ν_{10}	1391	12.9	1434	12.8	1435	12.4
ν_{11}	1346	3.0	1384	3.5	1384	3.0
ν_{12}	1276	4.6	1343	18.5	1338	7.4
ν_{13}	1180	44.4	1282	42.3	1280	40.8
ν_{14}	1146	7.0	1196	10.9	1234	9.4
ν_{15}	1081	38.1	1154	9.2	1127	21.4
ν_{16}	1030	23.7	1114	39.8	1106	34.0
ν_{17}	1000	8.5	1065	18.0	1061	7.6
ν_{18}	983	0.9	1045	0.6	1045	0.6
ν_{19}	939	11.4	1003	3.6	1012	6.0
ν_{20}	855	23.8	951	22.8	948	13.5
ν_{21}	743	21.6	862	16.0	860	35.9
ν_{22}	676	13.7	811	9.7	796	8.5
ν_{23}	563	2.3	582	35.5	566	6.6
ν_{24}	517	18.7	462	2.8	506	12.6
ν_{25}	411	6.9	427	8.8	432	10.5
ν_{26}	329	3.2	407	1.2	419	3.6
ν_{27}	270	3.9	344	4.9	330	5.1
ν_{28}	262	0.7	275	1.1	275	1.0
ν_{29}	247	0.4	209	0.4	217	1.0
ν_{30}	226	2.3	184	1.6	185	1.4
ν_{31}	212	0.4	122	1.4	145	1.6
ν_{32}	102	3.2	66	0.1	75	0.9
ν_{33}	79	3.2	41	2.4	34	2.1

mode	(*E*)-C(CH_3)ICHCH_2OO-1		(*E*)-C(CH_3)ICHCH_2OO-2		(*E*)-C(CH_3)ICHCH_2OO-3	
	ν^a/cm^{-1}	intensity	ν^a/cm^{-1}	intensity	ν^a/cm^{-1}	intensity
ν_1	3093	0.6	3093	0.9	3094	0.4
ν_2	3049	10.2	3059	7.1	3058	8.7
ν_3	3027	12.8	3032	12.0	3024	15.5
ν_4	3008	3.3	3008	3.6	3007	3.8
ν_5	2988	9.0	2992	17.0	2988	13.2
ν_6	2957	7.2	2958	7.0	2956	8.9
ν_7	1652	60.3	1650	52.1	1654	43.9
ν_8	1464	6.1	1459	7.9	1456	7.7
ν_9	1443	4.4	1438	4.2	1438	10.4
ν_{10}	1436	11.7	1434	10.5	1435	2.8
ν_{11}	1385	6.4	1386	6.1	1386	6.5
ν_{12}	1351	27.4	1346	10.5	1345	9.0
ν_{13}	1314	26.3	1312	14.4	1303	8.6
ν_{14}	1195	26.4	1239	15.2	1244	22.8
ν_{15}	1149	8.9	1126	25.2	1136	29.4
ν_{16}	1102	70.7	1106	59.9	1102	60.5
ν_{17}	1061	14.6	1053	4.2	1053	7.5
ν_{18}	1042	0.6	1042	0.6	1041	0.7
ν_{19}	966	5.1	984	12.4	981	8.5
ν_{20}	931	17.4	910	7.5	917	9.2
ν_{21}	875	17.8	872	26.0	856	14.8
ν_{22}	829	6.1	794	15.7	825	17.3
ν_{23}	610	10.6	612	16.9	609	19.9
ν_{24}	515	35.1	544	2.8	541	0.6
ν_{25}	447	1.8	442	11.9	440	9.5
ν_{26}	350	5.0	371	10.6	391	7.7
ν_{27}	305	0.6	318	1.7	290	1.6
ν_{28}	279	2.0	286	2.2	274	1.6
ν_{29}	211	0.2	213	0.1	215	0.9
ν_{30}	180	0.3	179	0.2	182	0.2
ν_{31}	90	1.9	130	0.4	121	0.4
ν_{32}	70	0.0	64	1.7	67	2.5
ν_{33}	43	3.1	50	2.6	53	1.7

[a]Harmonic vibrational wavenumber x scaled according to (0.9708 ± 0.0159) x + (9.3 ± 20.7); see text.

Table S4. Convolution of the Scaled Harmonic Vibrational Wavenumbers (in cm^{-1}) and IR Intensities (in km mol^{-1}) of Three Lowest-energy Conformers $C(CH_3)ICHCH_2OO$

mode	$C(CH_3)ICHCH_2OO$ conformers[a]			convoluted[b]
	$C(CH_3)ICHCH_2OO$-1	$C(CH_3)ICHCH_2OO$-2	$C(CH_3)ICHCH_2OO$-3	
ν_9	1439[c] (4.8)[d]		1439[c] (5.4)[d]	1439 (3.2)[d]
ν_9		1450[c] (6.1)[d]		1450 (2.2)
ν_{10}	1435 (12.9)	1434 (12.8)	1435 (12.4)	1434 (12.8)
ν_{11}	1384 (3.0)	1384 (3.5)	1384 (3.0)	1384 (3.2)
ν_{12}	1337 (4.6)	1343 (18.5)	1338 (7.4)	1342 (10.1)
ν_{13}	1272 (44.4)	1282 (42.3)	1280 (40.8)	1278 (43.1)
ν_{14}	1239 (7.0)		1234 (9.4)	1238 (4.7)
ν_{14}		1196 (10.9)		1196 (3.9)
ν_{15}		1154 (9.2)		1154 (3.3)
ν_{15}	1137 (38.1)		1127 (21.4)	1137 (21.7)
ν_{16}	1113 (23.7)	1114 (39.8)	1106 (34.0)	1113 (31.1)
ν_{17}	1057 (8.5)	1065 (17.9)	1061 (7.6)	1060 (11.7)
ν_{18}	1045 (0.9)	1045 (0.6)	1045 (0.6)	1045 (0.7)
ν_{19}	1015 (11.4)		1012 (6.0)	1015 (6.4)
ν_{19}		1003 (3.6)		1003 (1.3)
ν_{20}	936 (23.8)	951 (22.8)	948 (13.5)	941 (21.8)
ν_{21}		862 (16.0)	860 (35.9)	860 (13.4)
ν_{21}	837 (21.6)			837 (10.4)

[a]Predicted with the B3LYP/aug-cc-pVTZ-pp method. [b]Constructed from $C(CH_3)ICHCH_2OO$-1, $C(CH_3)ICHCH_2OO$-2, and $C(CH_3)ICHCH_2OO$-3 using their predicted Bolzmann population distribution of 48%, 36%, and 16%, respectively. [c]Harmonic vibrational wavenumber (cm^{-1}) scaled with $y = 0.9708\ x + 9.3$, in which x is the harmonic vibrational wavenumber. [d]IR intensities in km mol^{-1} are given in parentheses.

Table S5. Fitted Values of $I_{syn\text{-MVKO},0}$ and $I'_{syn-\text{MVKO},0}$ in N_2 and O_2 at Various Pressures

pressure /Torr	$I_{syn\text{-MVKO},0}$ (biexponential)[a]	$I'_{syn-\text{MVKO},0}$ (second-order)[b]	deviation
N_2 buffer gas			
14.9	0.081±0.007	0.082±0.007	0.001±0.010
20.0	0.086±0.006	0.085±0.008	−0.001±0.010
24.8	0.069±0.006	0.071±0.006	0.002±0.008
30.0	0.056±0.004	0.056±0.005	0.000±0.006
44.9	0.076±0.006	0.079±0.011	0.003±0.013
64.3	0.097±0.006	0.101±0.005	0.004±0.008
83.7	0.076±0.006	0.082±0.008	0.006±0.010
110.3	0.057±0.005	0.058±0.006	0.001±0.008
194.0	0.048±0.005	0.052±0.005	0.004±0.007
O_2 buffer gas			
31.7	0.086±0.004	0.104±0.005	0.018±0.006
63.8	0.080±0.003	0.087±0.012	0.007±0.012
106.6	0.050±0.002	0.047±0.005	0.003±0.005
190.2	0.032±0.002	0.033±0.005	0.001±0.005

[a]The temporal profiles were fitted using the biexponential equation $I_{syn\text{-MVKO}} = I_{syn\text{-MVKO},0} \times \frac{k_1}{k_1 - k_\mathrm{d}}[e^{-k_\mathrm{d}t} - e^{-k_1 t}$, in which $I_{syn\text{-MVKO}}$ is the integrated absorbance over 900–962 cm^{-1}. [b]The temporal profiles were fitted using the second-order rate equation $1/I_{syn\text{-MVKO}} = 1/I'_{syn-\text{MVKO},0} + 2k_{self}\, t$.

Table S6. Summary of Relative Yields (y_α^{rel}) and Estimated Absolute Yields (y_α) at Various Pressures

pressure (Torr)	y_α^{rel}	$1/y_\alpha^{\mathrm{rel}}$	y_α	$1/y_\alpha$	y_γ	$y_\alpha + y_\gamma$
N_2 buffer gas[a]						
14.9	0.59 ± 0.05	1.69 ± 0.14	0.23 ± 0.03	4.42 ± 0.52	0.16 ± 0.06	0.38 ± 0.06
20.0	0.47 ± 0.04	2.11 ± 0.19	0.18 ± 0.02	5.60 ± 0.60	0.20 ± 0.06	0.38 ± 0.05
24.8	0.44 ± 0.04	2.26 ± 0.21	0.17 ± 0.02	5.98 ± 0.69	0.21 ± 0.06	0.38 ± 0.06
30.0	0.46 ± 0.04	2.18 ± 0.19	0.16 ± 0.02	6.22 ± 0.67	0.19 ± 0.05	0.35 ± 0.05
44.9	0.38 ± 0.04	2.61 ± 0.25	0.15 ± 0.02	6.79 ± 0.77	0.24 ± 0.06	0.38 ± 0.06
64.3	0.33 ± 0.03	3.01 ± 0.29	0.13 ± 0.01	7.87 ± 0.80	0.26 ± 0.06	0.38 ± 0.05
83.7	0.27 ± 0.03	3.76 ± 0.49	0.10 ± 0.01	9.94 ± 1.39	0.28 ± 0.07	0.38 ± 0.07
110.3	0.19 ± 0.03	5.18 ± 0.82	0.07 ± 0.01	14.00 ± 2.24	0.30 ± 0.08	0.37 ± 0.08
194.0	0.12 ± 0.03	8.19 ± 1.99	0.04 ± 0.01	23.11 ± 5.43	0.31 ± 0.12	0.35 ± 0.12
O_2 buffer gas						
31.7	0.50 ± 0.03	2.01 ± 0.14	0.18 ± 0.01	5.65 ± 0.47	0.18 ± 0.04	0.36 ± 0.04
63.8	0.32 ± 0.03	3.08 ± 0.25	0.12 ± 0.01	8.65 ± 0.69	0.24 ± 0.04	0.36 ± 0.04
106.6	0.22 ± 0.02	4.61 ± 0.45	0.08 ± 0.01	12.0 ± 1.1	0.30 ± 0.05	0.38 ± 0.05
190.2	0.13 ± 0.02	7.67 ± 1.12	0.05 ± 0.01	20.8 ± 3.2	0.32 ± 0.08	0.37 ± 0.08

[a] Lin et al. (Phys. Chem. Chem. Phys. **2020**, 22, 13603) reported y_α^{rel} = 0.82, 0.70, 0.29, 0.33/0.26, 0.19/0.13, ~0.15, and 0.19 at 298 K and total pressures of 4.2, 7.6, 50.3, 101.4, 301.7, ~308, and 702.5 Torr.

Table S7. Experimental Conditions and Measurements of IR Intensities (in km mol^{-1}) of 1,3-diiodo-but-2-ene

P_T /Torr	period /s	flow rate [a] /cm^3 s^{-1}	ΔW [b] /mg	integration range (cm^{-1}) 1500−1400	1320−1265	1210−1120	1110−1030
18.4	1315	336.6	217.0	16.3[c]	20.3[c]	62.0[c]	18.1[c]
22.5	1254	275.3	218.6	17.1	20.3	62.0	19.6
36.3	1691	170.8	251.0	18.7	19.8	63.4	18.7
45.0	1035	238.6	155.9	16.8	20.9	67.0	16.4
71.3	1242	150.5	161.9	17.3	21.4	68.8	18.5
82.3	1278	130.2	170.2	18.2	20.9	66.5	18.0
average (km mol^{-1})				17.4±0.8	20.6±0.5	64.9±2.6	18.2±1.0
calculation[d] (km mol^{-1})				22.4	29.3	104.1	34.4
experiment/calculation				0.78±0.04	0.70±0.02	0.62±0.02	0.53±0.03

[a] Flow rate (in cm^3 s^{-1}) is at 298 K and P_T. [b] Total weight loss (in mg) over the listed period. [c] IR intensities (in km mol^{-1}) derived by measuring the integrated absorbance and ΔW according to equations S8−S10. [d] Predicted with the B3LYP/aug-cc-pVTZ-pp method.

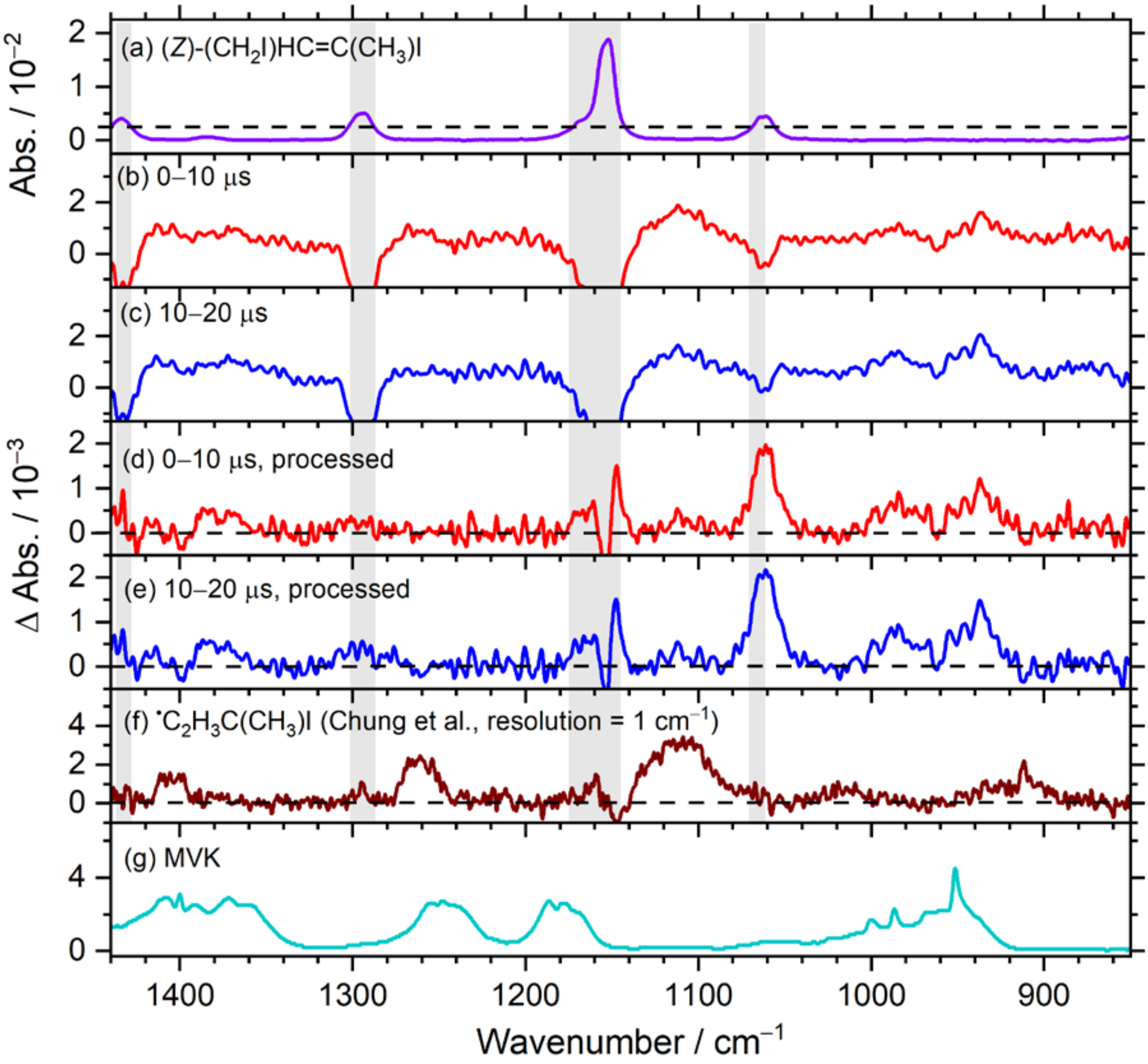

Figure S1. Observed and processed spectra in the region 1450−850 cm^{-1} upon photolysis at 248 nm of a flowing mixture of (*Z*)-(CH_2I)HC=C(CH_3)I/O_2/N_2 (0.05/15/179 Torr). The instrumental resolution is 2 cm^{-1}. An external ADC was employed. (a) Absorption spectrum before photolysis. Difference spectra recorded 0–10 μs (b) and 10–20 μs (c) after photolysis. (d) and (e): by removing bands of the radical precursor $^{\bullet}C_2H_3C(CH_3)I$ and methyl vinyl ketone (MVK), and adding back the decay of (*Z*)-(CH_2I)HC=C(CH_3)I. Gray areas indicate severe interference from the absorption of (*Z*)-(CH_2I)HC=C(CH_3)I. (f) Spectrum of radical precursor $^{\bullet}C_2H_3C(CH_3)I$ reported in Chung and Lee; reproduced or adapted with permission from reference 29 in the main text. Copyright 2021, http://creativecommons.org/licenses/by/4.0/, Springer Nature. (g) Reference spectrum of methyl vinyl ketone (MVK) at 15 Torr.

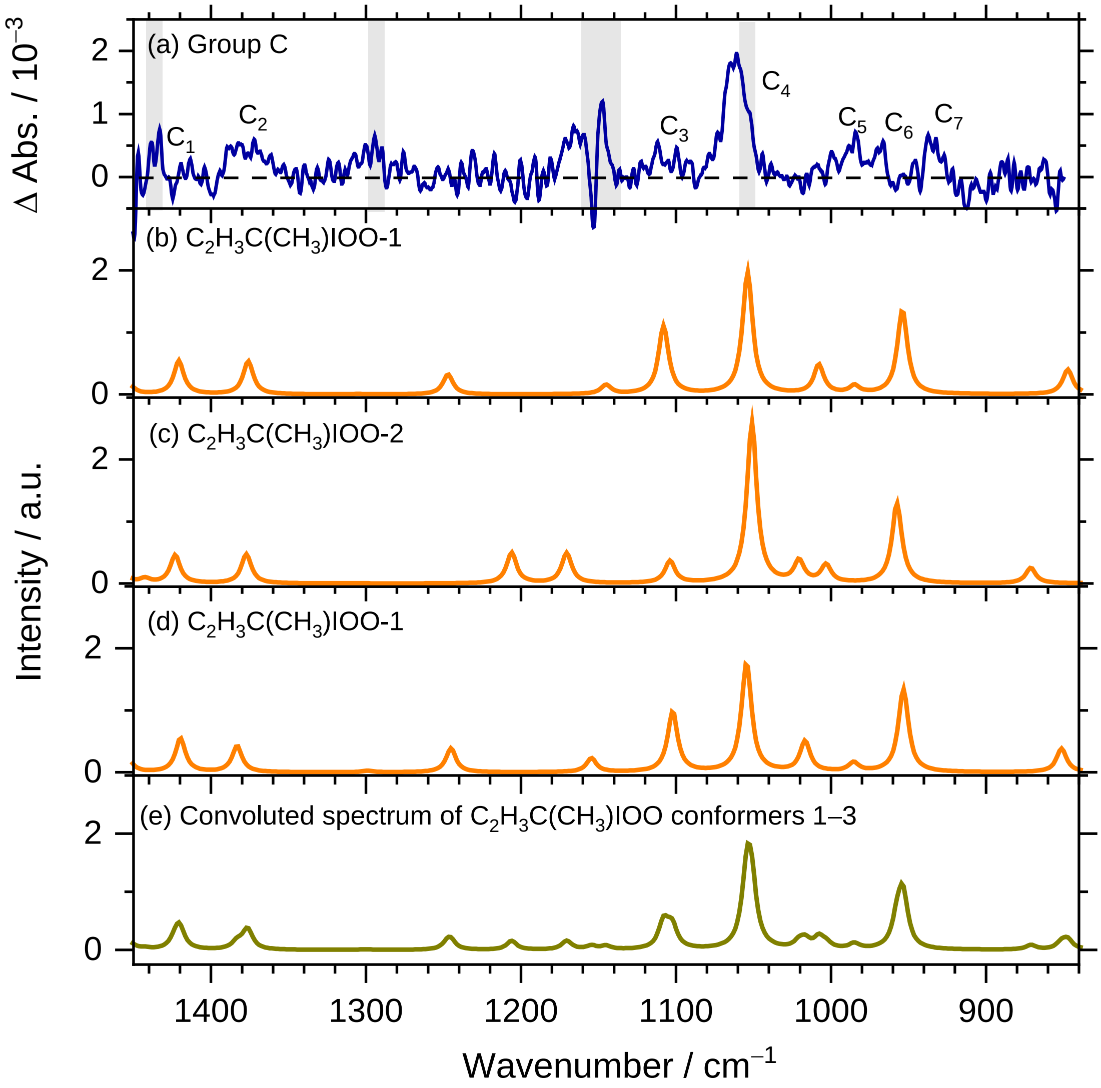

Figure S2. Convolution of spectra of three conformers of the adduct $C_2H_3C(CH_3)IOO$. (a) Processed spectrum of group C, taken from Figure 1f. Simulated spectrum of $C_2H_3C(CH_3)IOO$-1 (b), $C_2H_3C(CH_3)IOO$-2 (c) and $C_2H_3C(CH_3)IOO$-3 (d). (e) Boltzmann-weighted convoluted spectrum of three lowest-energy conformers $C_2H_3C(CH_3)IOO$ (conformer 1 : 2 : 3 = 40 : 31 : 29) at 298 K with a full width at half-maximum (FWHM) of 4 cm^{-1}.

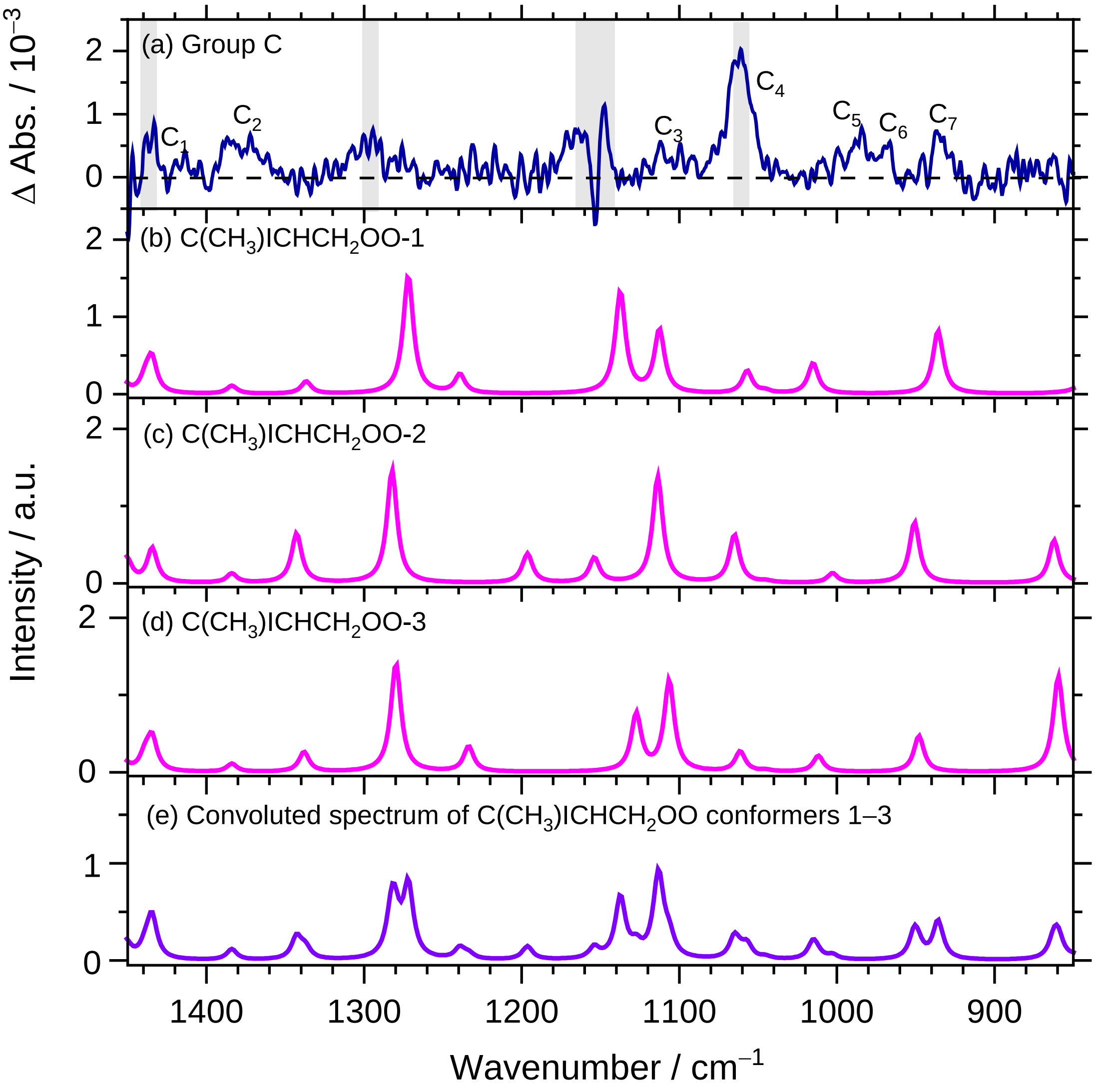

Figure S3. Convolution of three conformers of the adduct $C(CH_3)ICHCH_2OO$. (a) Processed spectrum of group C, taken from Figure 1f. Simulated spectrum of $C(CH_3)ICHCH_2OO$-1 (b), $C(CH_3)ICHCH_2OO$-2 (c) and $C(CH_3)ICHCH_2OO$-3 (d). (e) Boltzmann-weighted convoluted spectrum of three lowest-energy conformers $C_2H_3C(CH_3)IOO$ (conformer 1 : 2 : 3 = 48 : 36 : 16) at 298 K with a full width at half-maximum (FWHM) of 4 cm^{-1}.

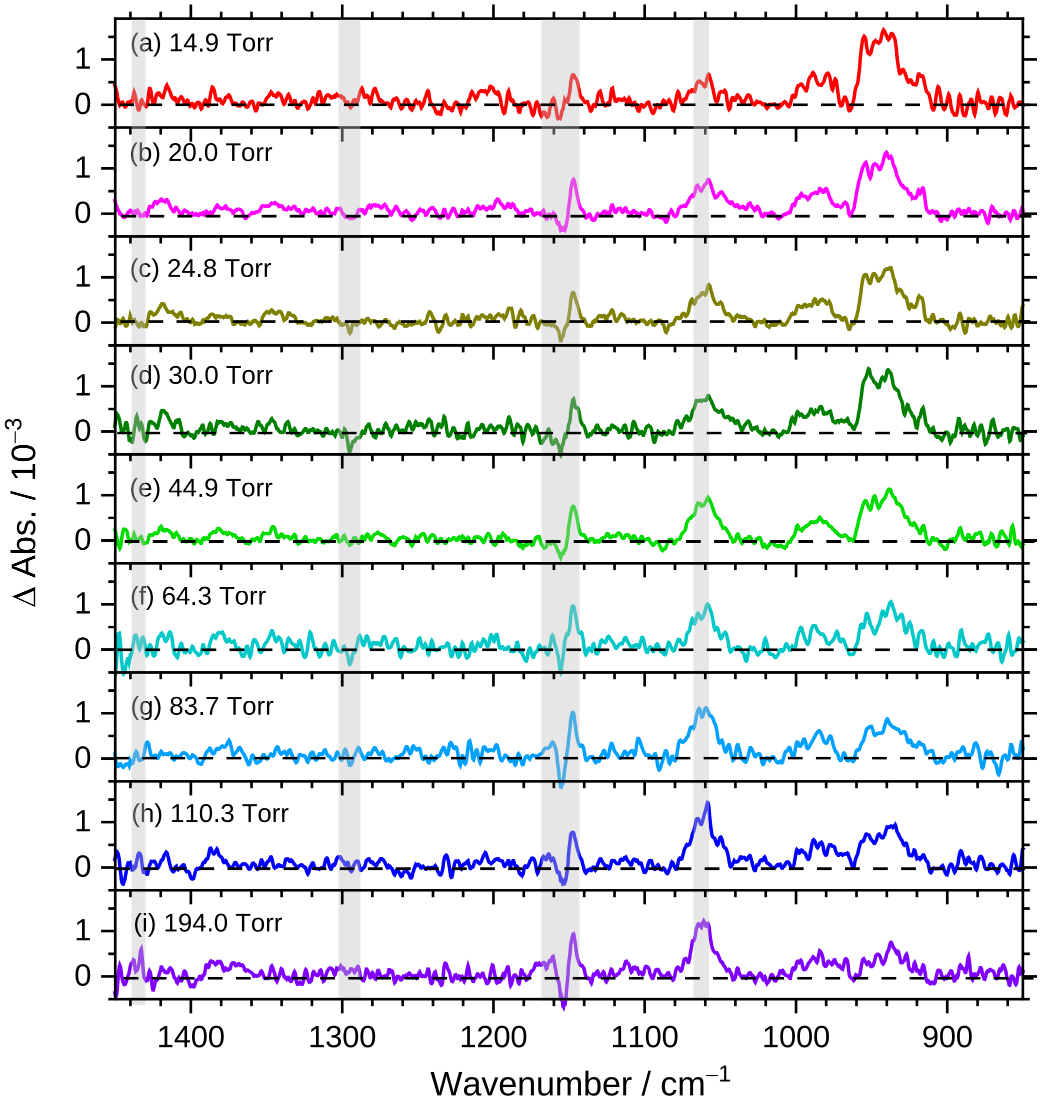

Figure S4. Processed spectra in the region 850–1450 cm^{-1} recorded 10–20 μs after photolysis at 248 nm of a flowing mixture of (*Z*)-(CH_2I)HC=C(CH_3)I/O_2/N_2 at various pressures. Spectra were recorded at total pressures P_T = 14.9 Torr (a), 20.0 Torr (b), 24.8 Torr (c), 30.0 Torr (d), 44.9 Torr (e), 64.3 Torr (f), 83.7 Torr (g), 110.3 Torr (h), and 194 Torr (i). The partial pressure of O_2 was maintained at 15 Torr in all experiments. Spectral resolution is 2 cm^{-1}.

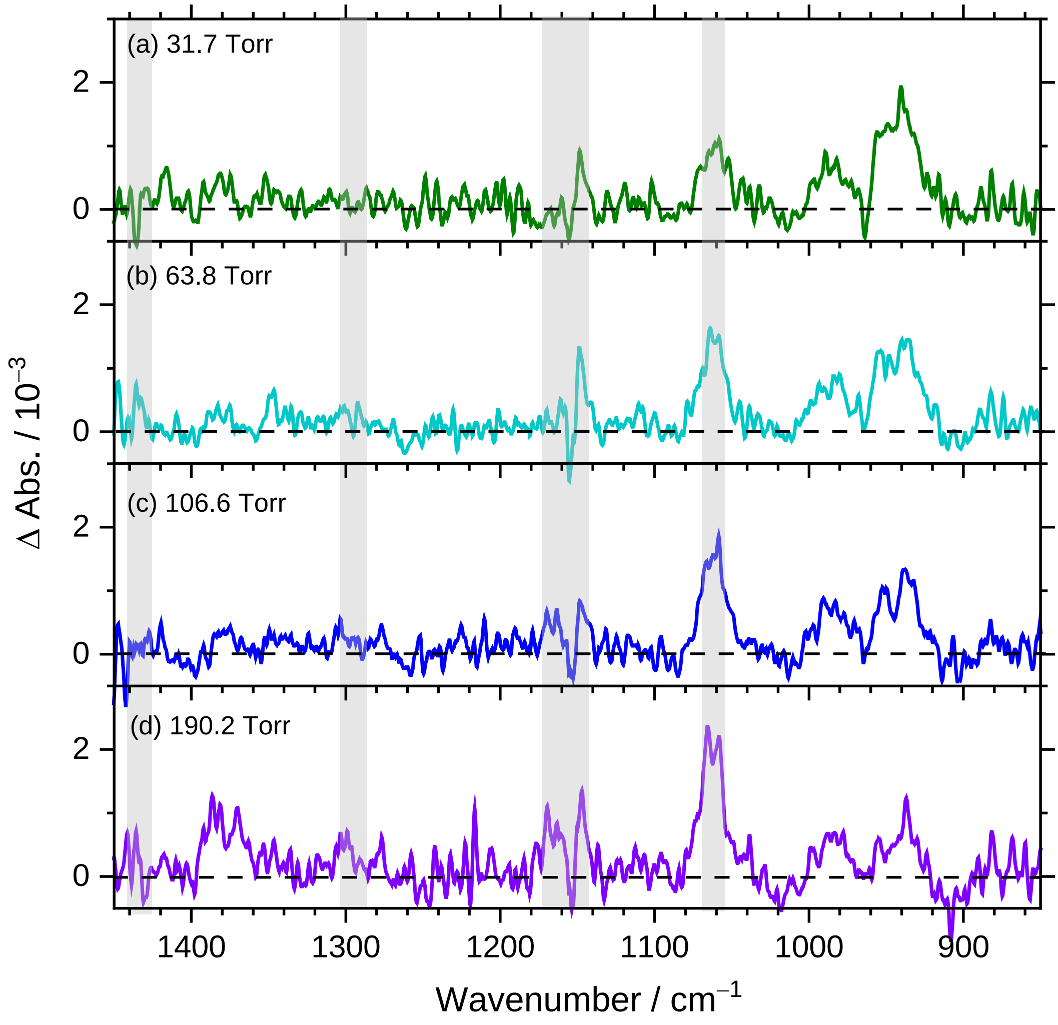

Figure S5. Processed spectra in the region 850–1450 cm^{-1} recorded 10–20 μs after photolysis at 248 nm of a flowing mixture of (*Z*)-(CH_2I)HC=C(CH_3)I/O_2 at various pressures. Spectra were recorded at total pressures P_T = 31.7 Torr (a), 63.8 Torr (b), 106.6 Torr (c), and 190.2 Torr (d). Spectral resolution is 2 cm^{-1}.

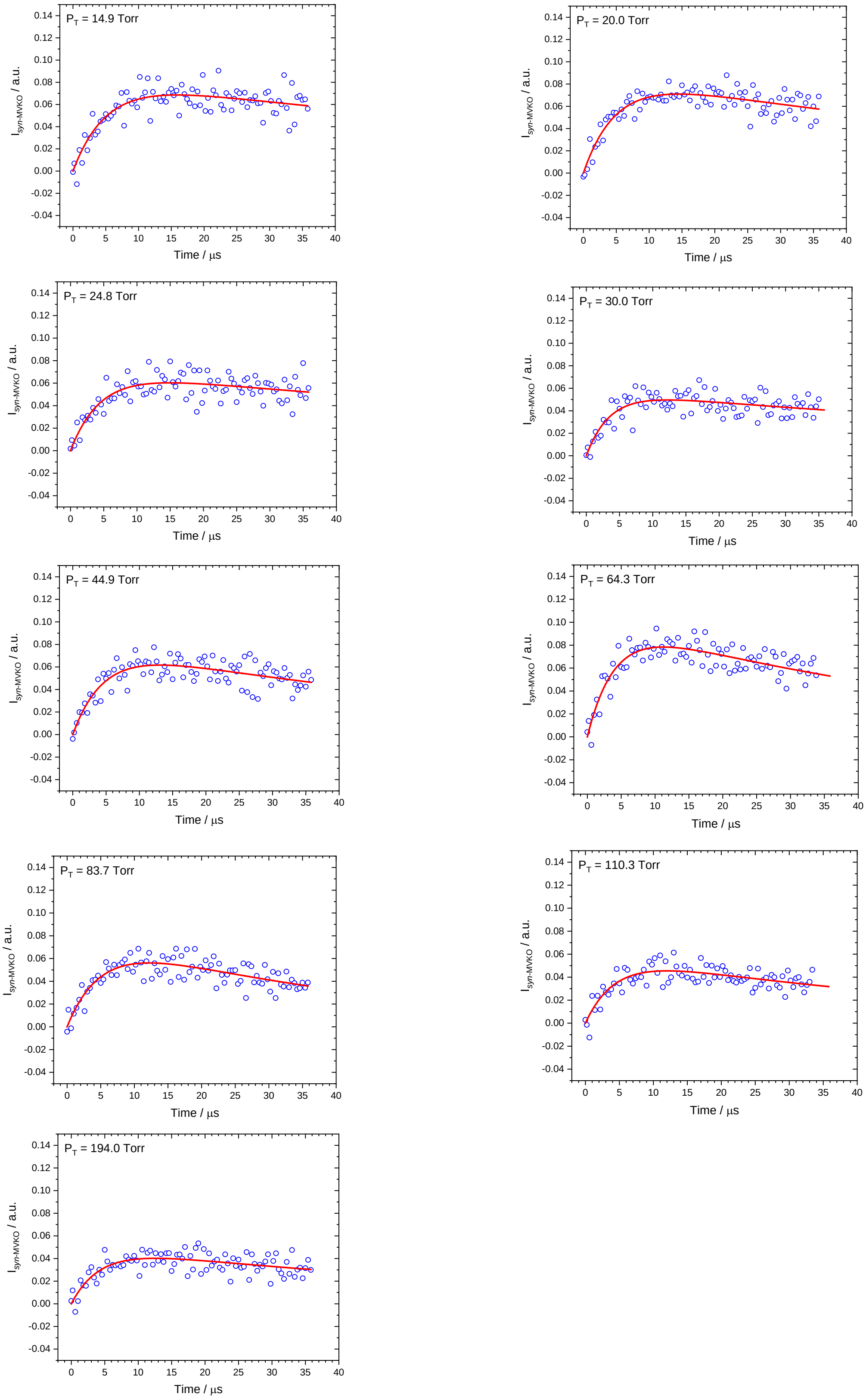

Figure S6. Temporal profiles of I_{syn}-MVKO in N_2 at various pressures. The data were fitted using the equation I_{syn}-MVKO = I_{syn}-MVKO,0 × $\frac{k_1}{k_1 - k_d}[e^{-k_d t} - e^{-k_1 t}]$, in which I_{syn}-MVKO is the absorbance of MVKO integrated over 900–962 cm^{-1} , k_1 is the formation rate coefficient, and k_d is the overall decay rate coefficient.

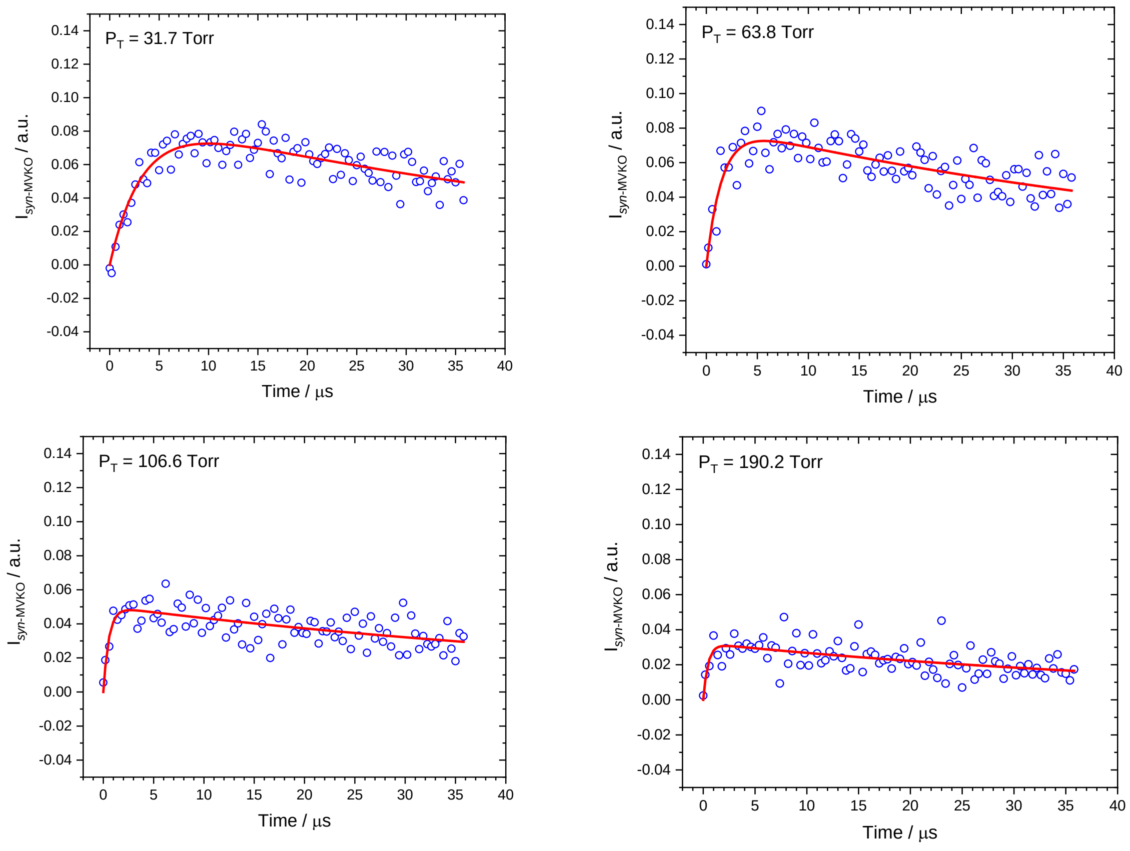

Figure S7. Temporal profiles of $I_{syn\text{-MVKO}}$ in O_2 at various pressures. The data were fitted using the equation $I_{syn\text{-MVKO}} = I_{syn\text{-MVKO},0} \times \frac{k_1}{k_1 - k_d}[e^{-k_d t} - e^{-k_1 t}]$, in which $I_{syn\text{-MVKO}}$ is the absorbance of MVKO integrated over 900–962 cm^{-1} , k_1 is the formation rate coefficient, and k_d is the overall decay rate coefficient.

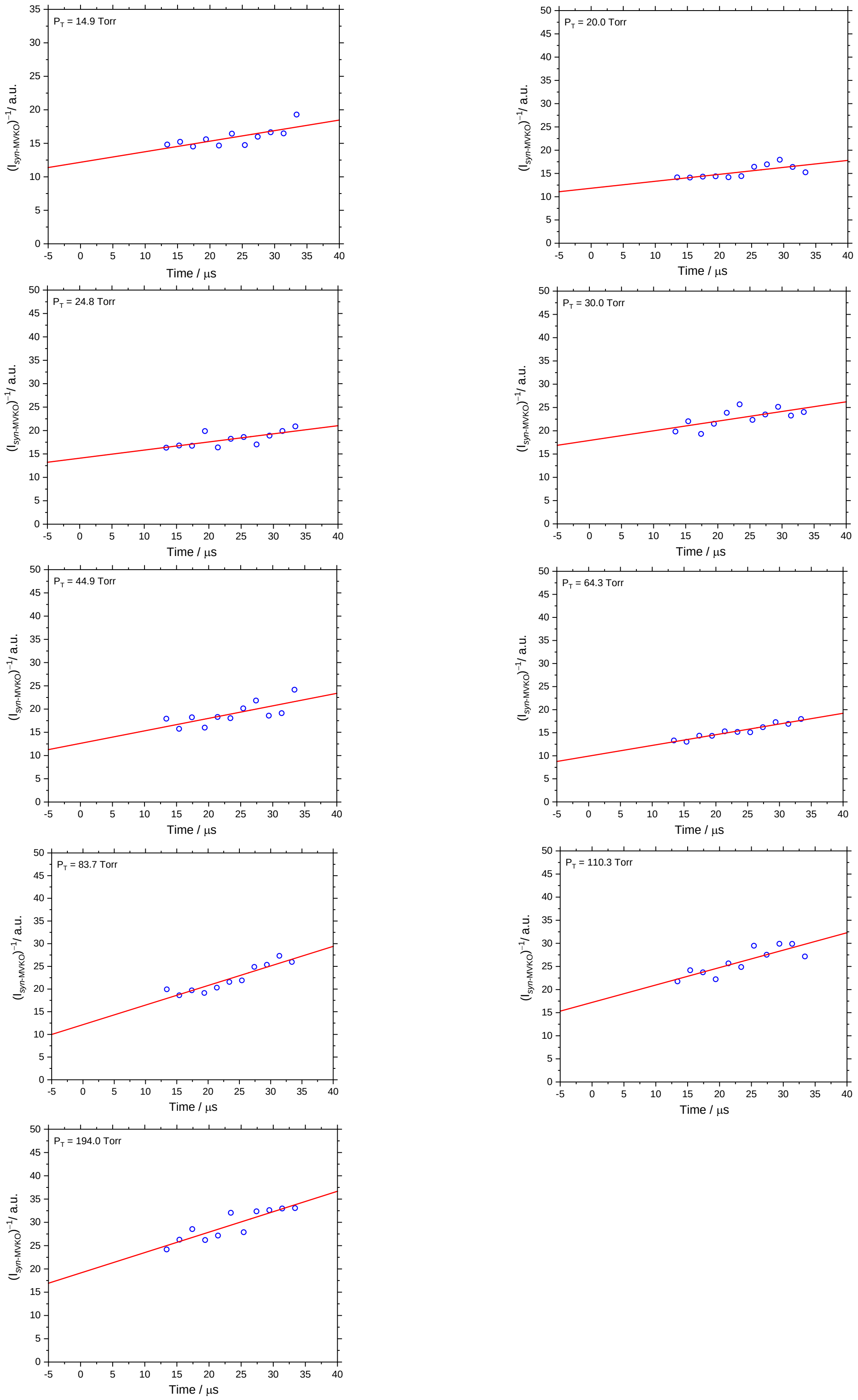

Figure S8. Temporal profiles of $1/I_{syn}$-MVKO in N_2 at various pressures. The data were fitted using equation $1/I_{syn}\text{-MVKO} = 1/I'_{syn-\mathrm{MVKO},0} + 2k_{self}\, t$, in which I_{syn}-MVKO is the absorbance of MVKO integrated over 900–962 cm^{-1} and k_{self} is the self-reaction rate coefficient.

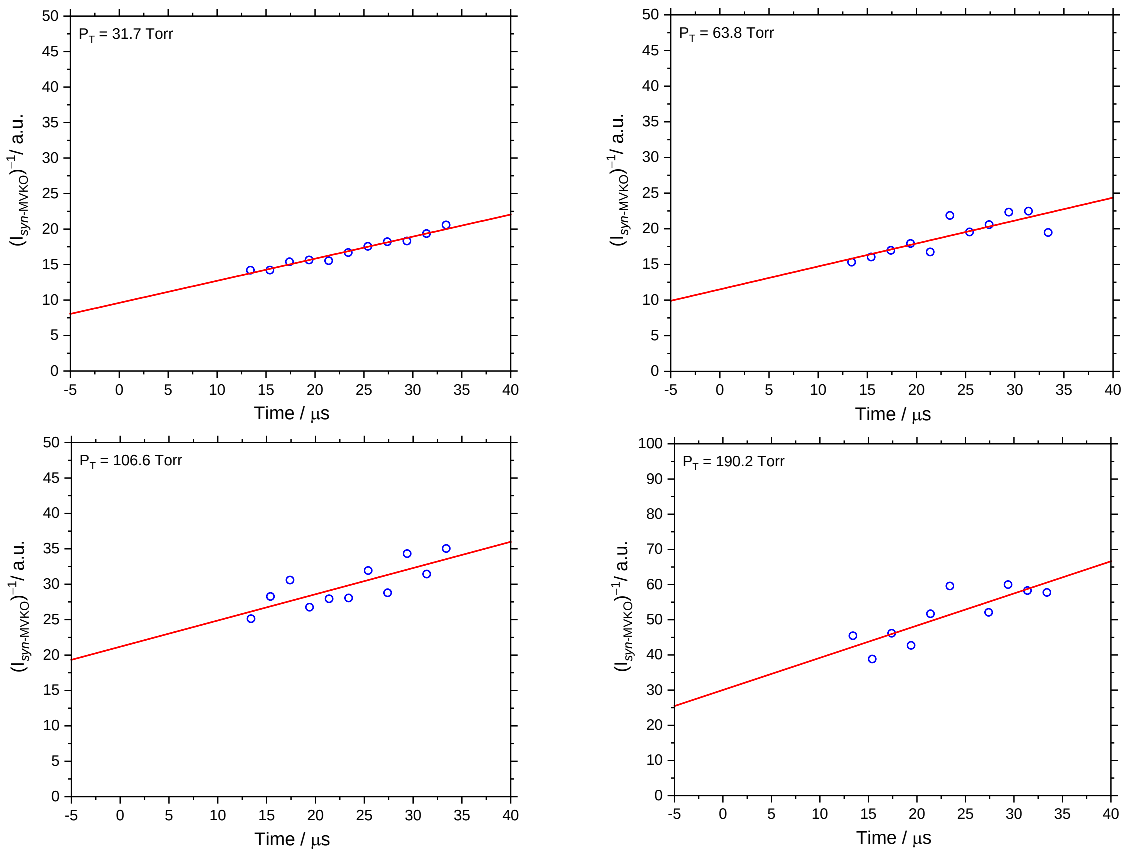

Figure S9. Temporal profiles of $1/I_{syn}$-MVKO in O_2 at various pressures. The data were fitted using the equation $1/I_{syn}$-MVKO = $1/I'_{syn-\mathrm{MVKO},0}$ + $2k_{self}\,t$, in which I_{syn}-MVKO is the absorbance of MVKO integrated over 900–962 cm^{-1} and k_{self} is the self-reaction rate coefficient.

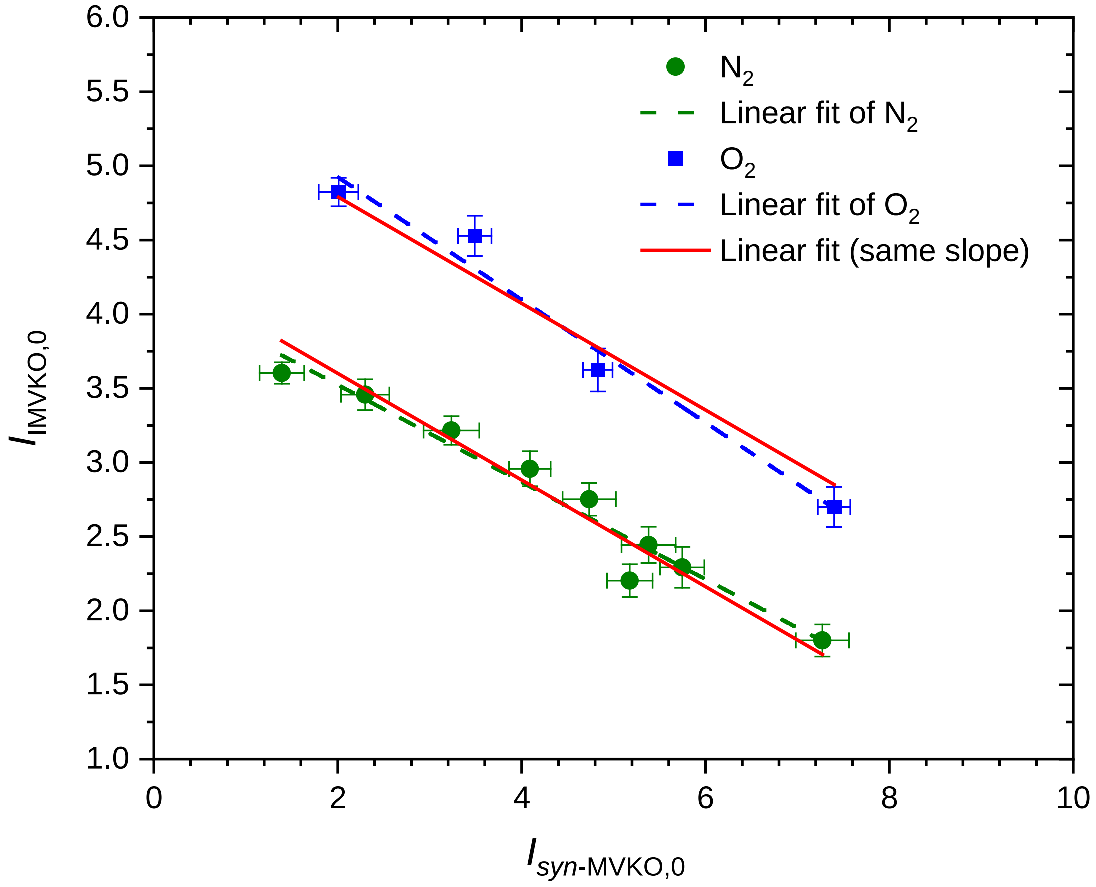

Figure S10. Plot of integrated absorbance of $C_2H_3C(CH_3)IOO$ ($I_{IMVKO,0}$) versus that of $C_2H_3C(CH_3)OO$ ($I_{syn\text{-}MVKO,0}$) at various pressures in two sets of experiments. $I_{IMVKO,0}$ was obtained on integrating the 1035–1085 cm^{-1} region of spectra recorded over 10–20 μs after photolysis. $I_{syn\text{-}MVKO,0}$ was obtained by extrapolating the temporal profiles of $I_{syn\text{-}MVKO}$, integrated across the 900–965 cm^{-1} region, to time zero using the consecutive-reaction model. The green circles and blue squares represent experiments in N_2 and O_2, respectively. The fitted dashed lines with slopes −0.33 ± 0.03 (green) and −0.41 ± 0.05 (blue) were obtained on fitting the data with a linear equation. In addition, the datasets were simultaneously fitted with a shared slope of −0.36 ± 0.03, shown as the red solid line.

Reference

1. Lin, Y.-H. *et al*. The role of the iodine-atom adduct in the synthesis and kinetics of methyl vinyl ketone oxide—a resonance-stabilized Criegee intermediate. *Phys. Chem. Chem. Phys.* **22**, 13603–13612 (2020).
